# Baobab (*Adansonia digitata* L.) Fruit Pulp in Nutritional Therapeutics: Mechanistic Insights, Clinical Evidence, and Future Research Priorities

**DOI:** 10.3390/foods15203579

**Published:** 2026-10-09

**Authors:** Trust Mukudzei Pfukwa, Mukhtar Iderawumi Abdulraheem, Adeyemi O. Aremu, Olaniyi A. Fawole

**Affiliations:** 1 Postharvest and Agroprocessing Research Centre, Department of Botany and Plant Biotechnology, University of Johannesburg, Auckland Park, Johannesburg 2006, South Africa; tmpfukwa@gmail.com (T.M.P.); m.iderawumi@gmail.com or mukhtara@uj.ac.za (M.I.A.); 2 South African Research Chairs Initiative in Sustainable Preservation and Agroprocessing Research, Faculty of Science, University of Johannesburg, P.O. Box 524, Auckland Park, Johannesburg 2006, South Africa; 3 Centre of Excellence in Indigenous Knowledge Systems, Faculty of Natural and Agricultural Sciences, North-West University, Private Bag X2046, Mmabatho 2745, South Africa; oladapo.aremu@nwu.ac.za; 4 South African Research Chairs Initiative in Indigenous Knowledge-Driven Medicinal Plants Utilisation and Conservation Strategies for Human, Animal, and Crop Health (IK-Medplants4HAC), Faculty of Natural and Agricultural Sciences, North-West University, Private Bag X2046, Mmabatho 2745, South Africa

**Keywords:** baobab fruit pulp, nutritional transition, gut homeostasis, symbiotic, indigenous fruit, immune system support

## Abstract

The renewed interest in indigenous African foods reflects a shift towards traditional, nutrient-dense alternatives to address the burden of non-communicable diseases and micronutrient deficiencies. This review evaluates the evidence for proposed health benefits of baobab (*Adansonia digitata* L.) fruit pulp (BFP) across four domains: gut homeostasis, immune modulation, glycemic control, and iron bioavailability. The evidence base is predominantly preclinical. Tier 3 (in vitro) and Tier 2 (animal) studies suggest that BFP fibers (low-methoxylated homogalacturonan, xylogalacturonan, and possibly inulin and fructooligosaccharides) may exert prebiotic-like effects by selectively stimulating Bifidobacteriaceae and Lactobacilli, while BFP polyphenols and organic acids have been associated with enzyme inhibition and enhanced iron bioaccessibility, respectively. The methodological quality of these studies was not assessed. However, human evidence is scarce. Only six human intervention studies were identified, limited to glycemic response in healthy normoglycemic adults and one iron status trial in schoolchildren, which showed increased hemoglobin but no significant improvement in ferritin or soluble transferrin receptor. No human studies have examined gut microbiota modulation, immune outcomes, or effects in diabetic or pre-diabetic populations. Consequently, clinical efficacy has not been established, and BFP cannot currently be characterized as having confirmed prebiotic or therapeutic properties. The current evidence supports potential prebiotic and enzyme-inhibitory activity in preclinical models only. Evidence-based dietary recommendations require high-quality human intervention trials with clinically meaningful endpoints, standardization of BFP preparations, and systematic investigation of real-world dietary contexts. Future research should address these gaps to advance the evidence base for BFP. Practical barriers to translation, including sensory acceptance, product adulteration, and sustainable sourcing, are briefly considered.

## 1. Introduction

The global burden of metabolic syndrome and non-communicable diseases (NCDs) continues to escalate, with low- and middle-income countries experiencing the most rapid increases [1,2]. Cardiovascular diseases, diabetes, and diet-related cancers now account for approximately 74% of all deaths worldwide, with unhealthy dietary patterns identified as the leading modifiable risk factor [3]. Concurrently, food systems face unprecedented challenges from climate change, biodiversity loss, and supply chain disruptions, necessitating urgent transformation toward more sustainable and resilient models [4,5]. Metabolic syndrome and non-communicable diseases, including cardiovascular diseases, diabetes, cancer, and chronic respiratory diseases, pose significant global health challenges [6,7]. They often occur as comorbidities, placing a significant burden on health systems worldwide [8]. Both health conditions share common risk factors, including unhealthy diets, physical inactivity, substance abuse, urbanization, an aging population, economic transition, and inequality. As diet is one of the key contributing factors to these health challenges, it is prudent to consider how food systems can be reevaluated to inform interventions that mitigate them [9].

Within this context, indigenous and traditionally underutilized food species have garnered renewed scientific attention as potential dual-purpose solutions for nutritional security and sustainable agriculture [10]. The current food system has undergone significant shifts in community environments, sociocultural influences, industrial influences, and governmental and global influences [11]. To a large extent, it is increasingly difficult for individuals to make healthy food choices. When this scenario is superimposed on the existing challenges of environmental sustainability, there is an urgent need to implement interventions within the existing food system [9].

In this regard, the search for sustainable and healthy food sources has seen growing interest in previously underutilized foods [12]. In the African context, many of these ‘underutilized’ plants serve as significant sources of human and animal food, income, and medicine [12,13]. Baobab fruit from the *Adansonia* tree is gaining prominence as a functional food ingredient and platform for reverse nutritional transition. Baobab fruit is predominantly found in Africa and is rich in essential nutrients, as highlighted by Chadere et al. [14], and more recently by Assogbadjo et al. [15], Braca et al. [16], Mohammed et al. [17], and Sokeng et al. [18]. These findings were supported by earlier research by Sodimu et al. [19], who evaluated the medicinal uses and composition of the baobab tree in the Zaria Local Government Area of Kaduna State, Nigeria. Various parts of the tree have been used to treat and prevent a host of ailments, including urinary tract infections, tuberculosis, anemia, diabetes, and malaria. Similarly, recent research by Offiah and Falade [20] has highlighted the potential for African baobab domestication and cultivation, as well as its nutrient variability, novel food applications, and industrial value. While they conceded that the fruit pulp currently carries more market value, mounting evidence points to broader industrial applications of other plant parts.

Globally, the European Commission’s decision to place baobab fruit pulp on the market as a novel food ingredient [21] has helped to shed its previously underutilized status. In that regard, Buchmann et al. [22] considered how the increase in the popularity and trade of baobab fruit can influence changes in power relations and access rights within rural households that rely on African baobab for their livelihoods. Furthermore, their work explored the elements critical to the sustainable and ethical trade of African baobabs, including certification and capacity building to empower local communities.

The significance of baobab fruit pulp (BFP) as a functional food ingredient is increasing [8], with several proposed health benefits emerging. These include its effects in maintaining gut homeostasis, boosting immunity, and ameliorating blood sugar levels and iron absorption. Previous works have highlighted the nutritional value of baobab food products in relation to their nutritional composition [13,14,21]. However, there is a dearth of mechanistic evidence to support or explain the link between nutritional elements in BFP and proposed health benefits. While Silva et al. [23] reviewed the benefits of BFP in glucose homeostasis, there is evidence that it forms part of the broader effect of BFP in providing interconnected benefits that synergistically improve overall well-being. The baobab tree exemplifies the paradigm of indigenous foods supporting African communities for millennia while recently gaining global recognition as a novel food ingredient [24]. However, translating traditional knowledge into evidence-based nutritional recommendations remains incomplete, with significant gaps in the mechanistic understanding of the health benefits attributed to baobab consumption. As such, this review explores the mechanistic evidence supporting these purported health benefits, which may inform the development of evidence-based dietary strategies. However, the current evidence base does not yet support therapeutic claims; rather, it provides a mechanistic framework for designing future clinical investigations.

Previous reviews have addressed various aspects of baobab’s nutritional and therapeutic potential, yet significant gaps remain. Assogbadjo et al. [15] provided a 20-year bibliometric analysis but lacked a systematic mechanistic synthesis. Silva et al. [23] focused on glycemic effects but primarily described enzyme inhibition without pathway-level analysis or evidence quality assessment. Asogwa et al. [13] and Offiah and Falade [20] provided broad overviews of nutrition and food applications but did not critically evaluate mechanistic evidence or distinguish between in vitro, animal, and human data. None of these reviews applied explicit evidence grading criteria, addressed the disconnect between in vitro and clinical findings, or integrated implementation science perspectives such as adulteration, sustainable sourcing, and consumer acceptance. This review focuses on the mechanistic evidence for proposed BFP health benefits. Practical barriers to translation, including sensory acceptance, product adulteration, and sustainable sourcing, are considered briefly in Section 5.2, but are not the primary focus of this review, and no systematic search was conducted for market, consumer, or supply chain the literature. Detailed treatment of these dimensions is available in the previous work [20,25,26].

This review makes three contributions to the baobab literature. First, it synthesizes molecular-level mechanisms, including the AMPK-PGC-1α, NF-κB, PI3K/Akt, and SGLT1 pathways—that link BFP components to physiological outcomes, whereas prior reviews have largely described health effects without pathway-level analysis [13,15]. Second, it distinguishes evidence derived from human intervention studies, animal models, and in vitro systems, and uses a descriptive, design-based hierarchy to organize this evidence, addressing the conflation of evidence types that characterizes previous reviews [23]. Third, it briefly considers practical barriers to translation, including adulteration, sustainable sourcing, and consumer acceptance, which are relevant to the extent to which promising preclinical findings can be applied in practice. These contributions provide a framework for evaluating the evidence base and prioritizing future research.

## 2. Methodology

A structured narrative review was conducted to synthesize the available evidence on BFP composition, mechanisms, and health effects. The review process was informed by PRISMA (Preferred Reporting Items for Systematic Reviews and Meta-Analyses) 2020 reporting principles but does not claim full PRISMA compliance, as a formal risk-of-bias assessment of included studies was not performed [27]. Searches were performed across four electronic databases: Scopus, PubMed, Google Scholar, and Web of Science. The initial search was conducted on 12 September 2024 and covered all records available in the databases up to that date. An updated search was performed on 15 May 2026 to capture literature published between September 2024 and May 2026, using the same four databases, identical search strings and field tags, and the same inclusion and exclusion criteria as the initial search. The only difference between the two searches was the extended coverage period; no changes were made to the search strategy or eligibility criteria. No language restrictions were applied during the database search; however, only studies with full texts accessible in English (or an English translation) were eligible for inclusion in the final search. The search strategy was designed to identify studies on BFP composition, mechanisms, and health effects. Literature on market trends, consumer behavior, certification schemes, and supply-chain economics was not systematically searched and is cited selectively for context only. The review should therefore be regarded as a synthesis of mechanistic and clinical evidence, with practical translation barriers addressed briefly in Section 5.2. Search strategies combined MeSH terms and keywords organized into four thematic clusters: (1) plant identity: “baobab” OR “*Adansonia digitata*” OR “*Adansonia*” AND “fruit pulp”; (2) nutritional composition: “nutrient” OR “mineral” OR “vitamin” OR “fiber” OR “polyphenol” OR “phytochemical”; (3) health outcomes: “gut health” OR “glycemic” OR “immune” OR “iron absorption” OR “antioxidant” OR “anti-inflammatory”; and (4) mechanisms: “mechanism” OR “pathway*” OR “bioaccessibility” OR “bioavailability.” The following search terms were used in various combinations: baobab fruit pulp, indigenous African foods, nutritional transition, gut health, gut homeostasis, synbiotic, prebiotic, probiotic, gut microbiota, polyphenols, pectic polysaccharides, immune system support, therapeutic interventions, food-to-food fortification, and nutritional security. Duplicate records were removed, and two independent reviewers screened titles and abstracts against predefined inclusion criteria: (a) original research or systematic reviews examining baobab fruit pulp composition or biological activities; (b) studies reporting quantitative data on human, animal, or in vitro models; and (c) publications in peer-reviewed journals. Exclusion criteria included: (i) studies focusing exclusively on other plant parts (leaves, bark, seeds, roots) without fruit pulp data; (ii) review articles, commentaries, or editorials without original data; (iii) studies published in non-peer-reviewed sources; and (iv) studies for which a full text in English (or an English translation) was not available, or which lacked sufficient data for extraction. Reference lists of included articles were hand-searched for additional relevant studies. Data extraction focused on study design, experimental models, dosages, health outcomes, and proposed mechanisms of action.

### 2.1. Search Strategy and Reproducibility

The search was conducted across four electronic databases on 15 May 2026. The exact search strings, search fields, and database-specific adaptations are provided below.

*Scopus (search fields: Title, Abstract, Keywords):*

TITLE-ABS-KEY((“baobab” OR “Adansonia digitata” OR “Adansonia”) AND (“fruit pulp” OR “fruit powder” OR “pulp”)) AND TITLE-ABS-KEY((“nutrient*” OR “mineral” OR “vitamin” OR “fiber” OR “fibre” OR “polyphenol” OR “phytochemical” OR “organic acid*” OR “ascorbic acid” OR “citric acid”)) AND TITLE-ABS-KEY((“gut health” OR “glycemic” OR “glucose” OR “immune” OR “immunomodulat” OR “iron” OR “bioaccessibility” OR “bioavailability” OR “antioxidant” OR “anti-inflammatory”)) AND TITLE-ABS-KEY((“mechanism*” OR “pathway” OR “molecular” OR “signaling” OR “in vitro” OR “in vivo” OR “clinical” OR “human” OR “animal” OR “cell” OR “fermentation”))—412 records retrieved.

*PubMed (search fields: Title/Abstract; MeSH terms applied where available):*

(“baobab”[Title/Abstract] OR “Adansonia digitata”[Title/Abstract] OR “Adansonia”[Title/Abstract]) AND (“fruit pulp”[Title/Abstract] OR “fruit powder”[Title/Abstract] OR “pulp”[Title/Abstract]) AND (“nutrient”[Title/Abstract] OR “mineral”[Title/Abstract] OR “vitamin”[Title/Abstract] OR “fiber”[Title/Abstract] OR “fibre”[Title/Abstract] OR “polyphenol”[Title/Abstract] OR “phytochemical*”[Title/Abstract] OR “organic acid*”[Title/Abstract] OR “ascorbic acid”[Title/Abstract] OR “citric acid”[Title/Abstract]) AND (“gut health”[Title/Abstract] OR “glycemic”[Title/Abstract] OR “glucose”[Title/Abstract] OR “immune”[Title/Abstract] OR “immunomodulat*”[Title/Abstract] OR “iron”[Title/Abstract] OR “bioaccessibility”[Title/Abstract] OR “bioavailability”[Title/Abstract] OR “antioxidant”[Title/Abstract] OR “anti-inflammatory”[Title/Abstract]) AND (“mechanism*”[Title/Abstract] OR “pathway”[Title/Abstract] OR “molecular”[Title/Abstract] OR “signaling”[Title/Abstract] OR “in vitro”[Title/Abstract] OR “in vivo”[Title/Abstract] OR “clinical”[Title/Abstract] OR “human”[Title/Abstract] OR “animal”[Title/Abstract] OR “cell”[Title/Abstract] OR “fermentation”[Title/Abstract])—298 records retrieved.

*Web of Science (search field: Topic, which includes Title, Abstract, Author Keywords, and Keywords Plus):*

TS=(“baobab” OR “Adansonia digitata” OR “Adansonia”) AND TS=(“fruit pulp” OR “fruit powder” OR “pulp”) AND TS=(“nutrient*” OR “mineral” OR “vitamin” OR “fiber” OR “fibre” OR “polyphenol” OR “phytochemical” OR “organic acid*”) AND TS=(“gut health” OR “glycemic” OR “glucose” OR “immune” OR “immunomodulat” OR “iron” OR “bioaccessibility” OR “bioavailability”) AND TS=(“mechanism” OR “pathway” OR “in vitro” OR “in vivo” OR “clinical” OR “human”)—180 records retrieved.

*Google Scholar (search field: full-text; limited Boolean functionality):*

(“baobab” OR “Adansonia digitata”) AND “fruit pulp” AND (“nutrient” OR “fiber” OR “polyphenol” OR “organic acid”) AND (“gut” OR “glycemic” OR “immune” OR “iron”) AND (“mechanism” OR “pathway” OR “in vitro” OR “in vivo” OR “clinical”)—357 records retrieved.

Screening procedure for Google Scholar: Because Google Scholar does not support field-restricted searching or export of complete record sets, the first 100 results per search string were screened by title and abstract, and the remaining results were screened by title only. Records deemed potentially relevant were then retrieved for full-text assessment. The same inclusion and exclusion criteria were applied as for the other databases.

Records retrieved per database: Scopus, 412; PubMed, 298; Google Scholar, 357; Web of Science, 180 (total, 1247 records). After removal of 355 duplicates, 892 records remained for title and abstract screening. Of these, 673 were excluded, leaving 219 full-text articles assessed for eligibility. A further 124 articles were excluded, yielding 95 studies included in the final synthesis (28 in vitro, 34 animal/ex vivo, 6 human intervention, and 27 compositional/analytical studies).

### 2.2. Evidence Grading Framework

To organize the evidence supporting proposed BFP health benefits, we used a descriptive evidence hierarchy solely on study design. This framework is an author-developed classification intended to summarize the available evidence base for this review; it does not constitute a formal quality assessment or certainty-of-evidence grading. Methodological quality (risk of bias, blinding, allocation concealment, statistical power) was not systematically assessed, and no validated critical appraisal tool (e.g., Cochrane RoB 2, SYSCLE) was applied. Informed by the Oxford Centre for Evidence-Based Medicine (OCEBM) 2011 Levels of Evidence and the Joanna Briggs Institute (JBI) Levels of Evidence for Effectiveness [28,29]. This framework is an author-developed classification intended to summarize the available evidence base for this review; it does not claim to replicate or substitute for validated critical appraisal instruments such as the Cochrane Risk of Bias tool or GRADE. The hierarchy is based solely on study design, without imposing fixed sample-size thresholds or combining design with quality assessment.

Tier 1—Human intervention studies (randomized controlled trials (RCTs)) and non-randomized controlled trials conducted in human participants.
Tier 2—Animal or ex vivo studies (controlled animal studies, such as rodent, fish, or other vertebrate models) and ex vivo human tissue or cell studies.
Tier 3—In vitro studies (cell culture experiments, enzyme inhibition assays, fermentation models, and biochemical assays).
Tier 4—Compositional and analytical studies (no direct biological activity assessment)

For studies reporting multiple lines of evidence (e.g., both in vitro and animal experiments), the highest applicable level was assigned. The overall evidence status for each health theme is described using purely descriptive statements that indicate the tier(s) of available evidence and the number of studies, without assigning qualitative labels such as “established” or “promising.” For example, evidence is described as “Tier 1 evidence available (n = X), quality not assessed” or “Tier 3 evidence only (n = X); no Tier 1 or 2 data identified.” It should be noted that methodological quality (e.g., risk of bias, blinding, allocation concealment, statistical power) was not systematically assessed in this review, as formal risk-of-bias assessment was beyond the scope of this narrative synthesis. The assigned tier reflects the study design only and cannot distinguish between a high-quality randomized trial and a randomized trial with a small sample size, inadequate blinding, or insufficient controls. The assigned tier should not be interpreted as a judgment of methodological quality or the certainty of the evidence. This limitation is acknowledged in Section 4.6.

### 2.3. Terminology: Iron Bioaccessibility and Bioavailability

In this review, “iron bioaccessibility” refers to the fraction of iron released from the food matrix during gastrointestinal digestion that becomes available for intestinal uptake, typically measured using in vitro simulated digestion models. “Iron bioavailability” refers to the fraction of ingested iron that is absorbed and either utilized or stored by the body, and is assessed in vivo through serum ferritin, hemoglobin, soluble transferrin receptor (sTfR), or stable isotope methods. In vitro Caco-2 cell models provide an approximation of intestinal uptake but do not measure true bioavailability. These terms are not interchangeable and are used consistently throughout this review.

## 3. Traditional Uses and Composition of Baobab Fruit Pulp

Medicinal plants play a significant role in human and animal health in Africa, with 80% of the population relying on traditional medicines for primary healthcare [30]. The African baobab (*Adansonia digitata* L.) is one of eight species in the genus *Adansonia*, distinguished by its pachycaul growth, palmate leaves, and distinctive fruit. Comprehensive morphological descriptions and geographical distribution of all eight species are available elsewhere [15,31]. Indigenous knowledge systems have long identified the biological properties of baobab, with all parts of the plant used as food and medicine in various African communities. However, this review focuses specifically on the BFP, as it has the strongest emerging evidence of health benefits and is the most commercially developed [21,24]. In that regard, the African baobab achieves its ‘all-purpose’ status, with all parts of the plant used as food and medicine. Traditional uses of BFP are well-documented across sub-Saharan Africa. The pulp is commonly consumed fresh or dried, and is used to prepare porridge, sauces, and beverages as a form of food fortification [13,32]. Ethnobotanical reports cite the use of BFP to relieve diarrhea and dysentery, which may be attributed to its soluble fiber content and mineral-replenishing properties [32]. The pulp has also been used as an antipyretic in some communities, though this specific application requires further verification through controlled studies [21]. Other parts of the baobab tree, including bark, leaves, and seeds, have traditional medicinal applications (e.g., wound healing, immune support) that have been reviewed elsewhere [13,21]. The nutritional value of the Baobab fruit is established by enhancing the nutritional profile of starchy foods that are part of many staple diets across Africa.

### 3.1. Morphology and Taxonomy

The baobab tree is a member of the genus *Adansonia* in the family Bombacaceae, which comprises 28 genera and approximately 200 species of trees [33]. The genus *Adansonia* consists of eight species of baobab: *Adansonia digitata* (African baobab) is endemic to sub-Saharan Africa, whereas *A. gregorii* (Australian baobab) is native to Australia. The remaining six species, including *A. grandidieri* (Grandidieri baobab), *A. perrieri* (Perrier baobab), *A. za* (Za baobab), *A. madagascariensis* (Madagascar baobab), *A. rubrostipa* (Fony baobab), and *A. suarezensis* (Suarez baobab), are native to Madagascar [31]. Key phenotypic traits differentiating these species include pachycaul growth, bark color and texture, leaf structure, flower morphology, and fruit characteristics. Comprehensive morphological descriptions of all eight species are available elsewhere [15,34]. Baobabs usually have distinctly thick trunks, which can be cylindrical or bottle-shaped, with constrictions at the top, where few branches appear [31]. The bark texture can range from smooth to warty, while bark color can be brownish, red, or gray. The deciduous nature of the baobab allows it to shed leaves in the dry season, whereas leafing and flowering commence in the wet season. The palmate arrangement of baobab leaves stems from the outward projection of leaflets from the petiole, resembling the palm of the hand [35]. The fruits formed were also variably shaped and textured, allowing for distinctions among species. According to Assogbadjo et al. [34], differences in tree height, number of branches, and fruit pod thickness were significantly correlated with patterns of genetic variation. This can account for the wide variation among baobab species within Madagascar across important phenotypic traits. Nevertheless, baobab populations in different climatic zones also show significant morphological differences within species, indicating the influence of abiotic factors [36].

### 3.2. Nutritional Composition

The value of African baobab fruit stems from its significant quantities of essential nutrients. Muthai et al. [37] determined the variations in the nutritional attributes of BFP in eastern, western, and southern Africa (Table 1). Crude fiber values ranged from 6.69–8.83 g/100 g (dry weight), with BFP from Kenya having the highest values [37]. The crude protein ranged from 1.86–2.24 g/100 g (*dw*), with BFP from Zambia and Kenya having the highest values. There was no significant difference in ash values among Tanzania, Kenya, Zambia, and Malawi, with content ranging from 3.36–4.41 g/100 g (*dw*). The analysis of mineral concentrations indicated the following order: K > Ca > Mg > P > Na > Cu > Fe > Zn > Mn.

Sokeng et al. [18] showed that the methanolic extracts of BFP from Cameroon were dominated by the presence of glucose, fructose isomers, and sucrose. Other sugar moieties identified included myo-inositol, α, β-galacturonic acid, and α-rhamnose. In contrast to Bougatef et al. [38], galacturonic acid was much less abundant (<1%) in the Cameroon samples. These differences can be attributed to the location of the trees (Mauritania vs. Cameroon) as well as the solvent used in the extraction (water vs. methanol).

The crude fiber composition of BFP was determined by Alba et al. [39], who identified a range of polysaccharides that were largely composed of linear galacturonans with xylose substitution, aptly termed xylogalacturonans. This was also confirmed by Debelo et al. [40], who found that it contained 54% xyloglucans (hemicellulosic) and 7% pectic glycosides. In particular, the pulp is rich in low-methoxylated homogalacturonan and contains inulin and fructooligosaccharides, as confirmed through high-performance anion exchange chromatography with pulsed amperometric detection (HPAEC-PAD) and nuclear magnetic resonance (NMR) spectroscopy [41,42]. Specifically, inulin content is estimated at 2.5–4.2% of total carbohydrates, while fructooligosaccharides (FOS) range from 1.8–3.5%, depending on provenance and extraction method [38,40]. These findings highlight its potential as a source of prebiotic substrates for gut microbiota modulation [42,43]. Patova et al. [44] conducted a structural analysis of pectin from BFP using DEAE-cellulose ion-exchange chromatography, HPSEC, monosaccharide analysis, and Nuclear Magnetic Resonance (NMR) spectroscopy. They determined that pectin found in BFP is composed of a repeating unit → 4)-α-GalpA-1-(1 → 4)-α-GalpA-(1→, with intermittent substitution at *O*-3 of galacturonic acid residues. The ratio of xylose:galacturonic acid subunits in the BFP xylogalacturonans ranged from 1:4 to 1:8, depending on the species and location of the baobab [44].

The tartness of BFP was attributed to its high organic acid content. High-field NMR spectroscopy of BFP from Cameroon indicated that citric acid was the most abundant at 77.3%, followed by malic acid, ascorbic acid, and gallic acid at 17.6%, 3.2%, and 1.3%, respectively. Other organic acids, including succinic acid, D-threo-isocitric acid, lactic acid, formic acid, and fumaric acid, were found in lower quantities, accounting for less than 0.34% of the organic acid content [18]. Values for titratable acidity for BFP from Tanzania were 8.8 g malic acid equivalent per 100 g dry weight, translating to a low pH observed for the same aqueous BFP extracts [44]. A higher range of titratable acidity (9.8–14.9 g citric acid, Eq./100 g) was observed by [45] for BFP from Kenya. Recent provenance-specific analysis by Ahmed et al. [46] comparing baobab pulp from Sudan and Senegal revealed significant differences: protein (2.943 g/100 g) and ash (5.39 g/100 g) were higher in Sudanese samples, while dry matter (89.45%), fat (0.834 g/100 g), and pH (3.25) were higher in Senegalese samples. Calcium (3286 mg/kg) and potassium (19,072 mg/kg) concentrations were notably high in both provenances, reinforcing BFP’s potential as a mineral-rich food ingredient.

**Table 1:** Nutritional composition of Baobab fruit pulp.

Component	Content (Range)	Basis	Fiber Fraction/Analytical Method	References
Macronutrients				
Moisture	8.82–13.81%	Fresh weight	–	[13,37,45,47,48,49]
Protein	1.58–3.24 g/100 g	Dry weight	Kjeldahl (N × 6.25)	[13,37,45,47,48,49]
Fiber (crude)	6.35–9.61 g/100 g	Dry weight	Weende method (acid/alkali digestion)	[37,48,49]
Fiber (TDF)	45.8–56.62 g/100 g	Dry weight	AOAC 985.29 (enzymatic-gravimetric)	[13,40,45,47]
Soluble fiber	Not quantified separately	–	–	–
Insoluble fiber	Not quantified separately	–	–	–
Ash	3.40–6.60 g/100 g	Dry weight	Muffle furnace (550 °C)	[13,37,45,47,48,49]
Total carbohydrates	22.9–81.35 g/100 g	Dry weight	Calculated by difference	[13,37,45,47,48,49]
Fat	0.21–0.70 g/100 g	Dry weight	Soxhlet extraction	[13,37,45,47,48,49]
Vitamins				
Ascorbic acid (Vitamin C)	163.8–288.9 mg/100 g	Dry weight	Titrimetric/HPLC	[45,47]
Minerals (mg/100 g dry weight)				
Calcium (Ca)	180–430 mg/100 g	Dry weight	Atomic absorption spectroscopy (AAS)/ICP-OES	[37,45,48,49]
Potassium (K)	1330–2390 mg/100 g	Dry weight	AAS/ICP-OES	[37,45,48,49]
Magnesium (Mg)	90–191 mg/100 g	Dry weight	AAS/ICP-OES	[37,45,48,49]
Phosphorus (P)	56.1–124 mg/100 g	Dry weight	Colorimetric/ICP-OES	[13,45,48]
Sodium (Na)	7.0–31.1 mg/100 g	Dry weight	AAS/ICP-OES	[45,48]
Iron (Fe)	1.2–9.3 mg/100 g	Dry weight	AAS/ICP-OES	[37,45,48,49]
Zinc (Zn)	0.7–2.9 mg/100 g	Dry weight	AAS/ICP-OES	[37,45,48]
Copper (Cu)	0.53–1.6 mg/100 g	Dry weight	AAS/ICP-OES	[37,45]
Sulfur (S)	1864–2164 ppm (mg/kg)	Dry weight	ICP-OES	[37]
Chlorine (Cl)	31,997–38,273 ppm (mg/kg)	Dry weight	ICP-OES	[37]

Footnote: Values represent ranges from multiple studies and geographical provenances. Crude fiber (Weende method) and total dietary fiber (AOAC enzymatic-gravimetric) are not directly comparable, as crude fiber underestimates true dietary fiber content due to loss of soluble fiber fractions during acid/alkali digestion. Values represent ranges from multiple studies and provenances. Detailed provenance-specific data are available in the original sources [21,37,45,47,48,49]. SDF = soluble dietary fiber; IDF = insoluble dietary fiber; TDF = total dietary fiber; dw = dry weight; nd = not determined.

#### Interpretation of Fiber Composition and Sources of Variation

The apparent discrepancy in fiber content reported in Table 2 (ranging from 6.35–9.61 g/100 g for crude fiber versus 45.8–56.62 g/100 g for total dietary fiber) reflects fundamental differences in analytical methodology rather than true compositional variation. Crude fiber, determined by the Weende method (sequential acid and alkali digestion), measures only the indigestible residue consisting primarily of cellulose and lignin, while solubilizing and discarding hemicellulose and pectin fractions [43]. By contrast, the AOAC enzymatic-gravimetric method (AOAC 985.29) measures total dietary fiber, which includes soluble fibers (pectins, gums, inulin, fructooligosaccharides) and insoluble fibers (cellulose, hemicellulose, lignin) that are not captured by crude fiber analysis [43]. As noted above, the choice of method (Weende vs. AOAC) has the most pronounced effect on reported values. Studies reporting “fiber” without specifying the method [32,41,42] should be interpreted with caution, as crude fiber values cannot be directly compared with total dietary fiber values.

Muthai et al. [37] reported that BFP from Kenya exhibited the highest crude fiber values (8.83 g/100 g dw), while samples from Tanzania and Malawi showed lower values (6.69–7.45 g/100 g dw). These differences may reflect variations in soil composition, rainfall patterns, and temperature during fruit development, which influence cell wall polysaccharide biosynthesis. The stage of fruit ripening at harvest significantly influences pectin composition and degree of methoxylation, as pectin is progressively de-esterified and solubilized during ripening. Alba et al. [39] reported differences in xylogalacturonan content between early- and late-harvested fruits, though systematic studies on maturity effects are lacking. Drying temperature, grinding intensity, and particle size affect the extractability and quantification of dietary fiber. Stadlmayr et al. [45] observed that fine-milled BFP powder yielded higher measured fiber content than coarse-milled samples, likely due to increased surface area for enzymatic digestion. The wide genetic variation among baobab populations across Africa [15] may contribute to differences in fiber composition, as polysaccharide biosynthesis is genetically controlled. However, systematic studies correlating genetic markers with fiber composition are currently unavailable.

### 3.3. Phenolic Composition of Fruit Pulp

The phenolic composition of BFP has been characterized using multiple analytical approaches, including HPLC-DAD, LC-MS/MS, UHPLC-QTOF-MS, and NMR spectroscopy. The major phenolic compound classes identified include flavonols (quercetin, rutin, tiliroside), flavanols (catechin, epicatechin, and proanthocyanidin dimers B1/B2 and trimer C1), hydroxycinnamic acid derivatives (chlorogenic, ferulic, caffeic acids), iridoid glycosides (verm inside, specioside), phenylethanoid glycosides (acteoside, isoacteoside), benzoic acid derivatives (gallic, protocatechuic acids), and coumarins [16,17,18,50,51,52]. A systematic summary of these compounds, their concentrations where available, analytical methods, and corresponding references is provided in Table 2.

Flavonols and flavanols represent the most consistently reported phenolic classes across studies. Braca et al. [16] identified 15 compounds in Malian BFP extracts, with tiliroside as the predominant flavonol glycoside. Proanthocyanidins were present as dimers (procyanidins B1, B2) and trimers (procyanidin C1), along with monomers of catechin and epicatechin [15,17,44]. Sokeng et al. [18] confirmed the presence of dimeric and trimeric proanthocyanidins and tiliroside in Cameroonian BFP extracts. Catechin and epicatechin were identified as the most prominent phenolic components in NMR-based metabolomic fingerprinting of Sudanese BFP [50]. Hydroxycinnamic acid derivatives, iridoid glycosides, and phenylethanoid glycosides have been identified in hydroethanolic extracts. Li et al. [52] identified four hydroxycinnamic acid glycosides, six iridoid glycosides (predominantly derived from catalpol and ajugol), and three phenylethanoid glycosides, noting that the presence of iridoid glycosides is of chemotaxonomic significance for the genus *Adansonia*. The total phenolic content (TPC) of BFP, measured by the Folin–Ciocalteu assay, ranges from 15.6 to 28.4 mg GAE/g dry weight, depending on extraction solvent and geographical provenance [16,17,50,51,53,54]. High-resolution LC-MS/QTOF analysis by Ismail et al. [51] revealed that baobab fruit shells contain significantly higher phenolic content than seeds and pulp, with quercetin and rutin as major compounds, supporting the valorization of baobab byproducts for nutraceutical applications [53,55].

**Table 2:** Summary of phenolic compounds identified in baobab *Adansonia digitata* L. fruit pulp.

Compound Class	Representative Compounds	Concentration Range	Analytical Method(s)	Key References
Flavonols	Quercetin, quercetin glycosides, rutin (quercetin-3-O-rutinoside), kaempferol, tiliroside (kaempferol-3-O-glucoside)	Quercetin: not quantified; Tiliroside: major component	HPLC-DAD, LC-MS/MS, UHPLC-QTOF-MS	[16,17,51,55]
Flavanols (monomers)	Catechin, epicatechin, gallocatechin	Catechin/epicatechin: major phenolic components (NMR fingerprinting)	HPLC-DAD, LC-MS/MS, NMR	[17,18,50,52]
Flavanols (oligomers)	Procyanidin B1, B2 (dimers); Procyanidin C1 (trimer); higher oligomers (DP ≥ 3)	Procyanidin B2: 0.5–2.0 mg/g; B1: 0.3–1.2 mg/g; C1: 0.2–0.8 mg/g (estimated from MS data)	HPLC-DAD, LC-MS/MS, MALDI-TOF	[16,18,53,56]
Hydroxycinnamic acid derivatives	Chlorogenic acid, ferulic acid, caffeic acid, p-coumaric acid, hydroxycinnamic acid glycosides (e.g., 1-O-(E)-caffeoyl-β-D-glucose, 6-O-(E)-caffeoyl-β-D-glucose)	Chlorogenic acid: 0.5–1.5 mg/g; Ferulic acid: 0.2–0.8 mg/g (estimated from MS data)	HPLC-DAD, LC-MS/MS, UHPLC-QTOF-MS, NMR	[17,18,50,52]
Iridoid glycosides	Verminoside, specioside, 6-O-(E)-feruloylcatalpol, 6-O-p-coumaroylajugol, 6-O-(E)-caffeoylajugol	Not quantified—major components in hydroethanolic extracts	NMR, LC-MS/MS	[52]
Phenylethanoid glycosides	Acteoside, isoacteoside, martynoside	Not quantified	NMR, LC-MS/MS	[52]
Benzoic acid derivatives	Gallic acid, protocatechuic acid, p-hydroxybenzoic acid, vanillic acid, syringic acid	Gallic acid: 0.1–0.5 mg/g	HPLC-DAD, LC-MS/MS	[17,50]
Coumarins	Furano-coumarins, pyrano-coumarins (tentatively identified)	Not quantified	UHPLC-QTOF-MS	[17,50]
Tannins	Hydrolyzable tannins (gallotannins, ellagitannins)	Not quantified separately (total tannins: ~2.5–5.0%)	Folin–Denis, HPLC	[13,17,54]
Total Phenolic Content (TPC)	–	15.6–28.4 mg GAE/g dw (depending on extract solvent and provenance)	Folin–Ciocalteu assay	[16,17,50,51,53,54]

Abbreviations: dw: dry weight; GAE: gallic acid equivalents; DP: degree of polymerization; HPLC-DAD: high-performance liquid chromatography with diode array detection; LC-MS/MS: liquid chromatography-tandem mass spectrometry; UHPLC-QTOF-MS: ultra-high-performance liquid chromatography-quadrupole time-of-flight mass spectrometry; NMR: nuclear magnetic resonance spectroscopy; MALDI-TOF: matrix-assisted laser desorption/ionization time-of-flight.

### 3.4. Distinction Between Baobab Fruit Pulp Preparations

Throughout this review, it is important to distinguish between different forms of baobab fruit pulp preparations, as they differ substantially in composition, bioactivity, and translational relevance. The whole baobab fruit pulp (BFP) is the unprocessed, dried fruit pulp, as consumed traditionally. Contains the full complement of fibers, polyphenols, organic acids, vitamins, and minerals in their native ratios. The dried and milled baobab fruit pulp (BFP powder) has a composition similar to that of the whole pulp, but particle size may affect bioavailability and fermentation kinetics. Most commercially available products and many studies use this form. Preparations are as follows: Extracts are obtained by extracting BFP with solvents (e.g., water, ethanol, methanol, acetone). Extracts are enriched in certain compound classes (e.g., polyphenols) and depleted in others (e.g., fibers) depending on the solvent and extraction conditions. Bioactivity may differ substantially from that of the whole pulp. Purified individual compounds (e.g., catechin, quercetin, procyanidin B2) can be derived from BFP or from other sources. These are used in mechanistic studies but do not represent the complexity of the whole BFP. Findings obtained with one preparation cannot be automatically extrapolated to others. For example, enzyme inhibition studies using methanolic extracts [17,57] may not fully reflect the effects of whole BFP consumption, where fibers and other matrix components may modulate polyphenol bioaccessibility and activity. Similarly, studies using isolated compounds from other sources [58,59] provide mechanistic hypotheses but do not constitute direct evidence for BFP-specific effects. Throughout this review, we have specified the preparation used in each study and interpreted findings accordingly.

### 3.5. Sources of Compositional Variability in BFP

The composition of BFP varies substantially across geographic origins, processing methods, and storage conditions, with direct implications for the reproducibility of biological effects reported across studies.

#### 3.5.1. Geographical Origin

Provenance-specific variation in BFP composition has been documented across African regions. Muthai et al. [37] reported significant differences in crude fiber (6.69–8.83 g/100 g dw), protein (1.86–2.24 g/100 g dw), and mineral concentrations across eastern, western, and southern African provenances. Stadlmayr et al. [45] observed that the ascorbic acid content ranged from 163.8 to 288.9 mg/100 g dw, depending on the geographical origin in Kenya. Similarly, total phenolic content varies from 15.6 to 28.4 mg GAE/g dw across provenances [16,17,50]. These variations are attributed to differences in soil composition, climatic conditions (temperature, rainfall), and genetic diversity among baobab populations [15,36].

#### 3.5.2. Processing Methods

Drying temperature, grinding intensity, and extraction solvent significantly affect BFP composition and bioactivity. High-temperature drying (>60 °C) can degrade heat-labile compounds, particularly ascorbic acid and certain polyphenols [45]. Particle size influences the extractability and bioaccessibility of bioactive compounds, with finer milling generally increasing measured fiber and polyphenol content [45,60]. Extraction solvent polarity determines the profile of recovered compounds: aqueous extracts retain water-soluble compounds (organic acids, sugars, some polyphenols), while organic solvents (methanol, ethanol, acetone) preferentially extract polyphenols and other lipophilic compounds [17,51,53]. These differences explain why studies using different extraction protocols may report divergent bioactivity profiles.

#### 3.5.3. Storage Conditions

Storage temperature, humidity, and duration affect BFP stability. Ascorbic acid is particularly susceptible to oxidative degradation during storage, with losses of up to 30–50% reported under ambient conditions over 6–12 months [47]. Advanced postharvest preservation technologies, including nanoparticle-essential oil formulations, have been developed to extend the shelf-life and maintain the quality of fruit-derived ingredients during storage [61,62,63]. Polyphenols may also undergo oxidation and polymerization during prolonged storage, potentially altering bioactivity. Moisture uptake during storage can promote microbial growth and enzymatic degradation, affecting overall product quality [20]. These factors underscore the importance of standardized storage conditions for both research and commercial applications.

#### 3.5.4. Implications for Biological Effects

The compositional variability described above has direct implications for the interpretation of biological effects reported across studies. Differences in ascorbic acid content may explain variations in iron bioaccessibility findings [64,65,66,67]. Variation in polyphenol profiles may contribute to differences in enzyme inhibition potency reported for glycemic studies [17,57]. Inconsistent fiber composition may affect prebiotic activity outcomes [42,43]. Without detailed compositional characterization of BFP batches used across different studies, it is difficult to distinguish genuine biological variation from raw-material variability. This underscores the need for standardized reporting of BFP provenance, processing conditions, and compositional analysis in future studies.

## 4. Health Benefits of Baobab Fruit Pulp and Mechanisms of Action

The popularity and interest in BFP powder are driven by several proposed health benefits to support its use [13,19]. Although indigenous knowledge and ethnomedicinal applications across Africa support these uses [19,21,22], several studies have investigated these purported effects [8,23,53,68]. The most important of these purported benefits include modulation of blood sugar levels, promotion of gut health, support of the immune system, and improvement of iron absorption (Table 3, Table 4, Table 5 and Table 6). Evidence supporting these purported effects, therefore, needs to be critically evaluated for the benefit of the growing baobab market and its health-conscious consumers.

### 4.1. Contribution to Gut Homeostasis

The high density of the microbial community in the colon and changes in its metabolites are of particular interest because they significantly influence host physiology. Gut ecology is modulated by an array of factors experienced by the host, including genetic predisposition, lifestyle, age, antibiotics, and the diet of the host [80,81]. Evidence shows that gut ecological communities play an important role in the overall health of the host by modulating the risk of metabolic syndrome disorders, including inflammation and obesity [80]. As such, diet provides significant therapeutic utility in altering the gut ecology toward a more beneficial composition for the host’s health. The mechanism by which the host structures gut microbiota composition depends on the ability of the host to control luminal electron acceptor availability and, in turn, oxygen availability in the intestines [82]. In that regard, the host diet facilitates or impairs intestinal cellular activities that shape the microbial environment [80,83].

In vitro and animal studies suggest a potential role for BFP in maintaining gut homeostasis [42]. Regardless of the wide variation in microbiota composition among individuals, BFP provides a way that allows the host to control microbiota composition in a way that sustains health. In support of this, Whiteman et al. [84] showed that substrate blends of polyphenols and fermentable fibers positively influenced the gut microbiota composition and metabolic function in healthy human subjects. Independently, the blends showed a selective positive impact on the gut state, whereas mixed substrate blends exerted a broader positive influence on the gut. Therefore, it is pertinent to elaborate on how BFP facilitates host control over microbiota composition. Firstly, through the provision of exogenous fermentable substrates, and secondly, through the polyphenol action on epithelial mitochondrial function [82,83].

#### 4.1.1. Effect of Baobab Fruit Fiber on Gut Function

**Figure 1:**
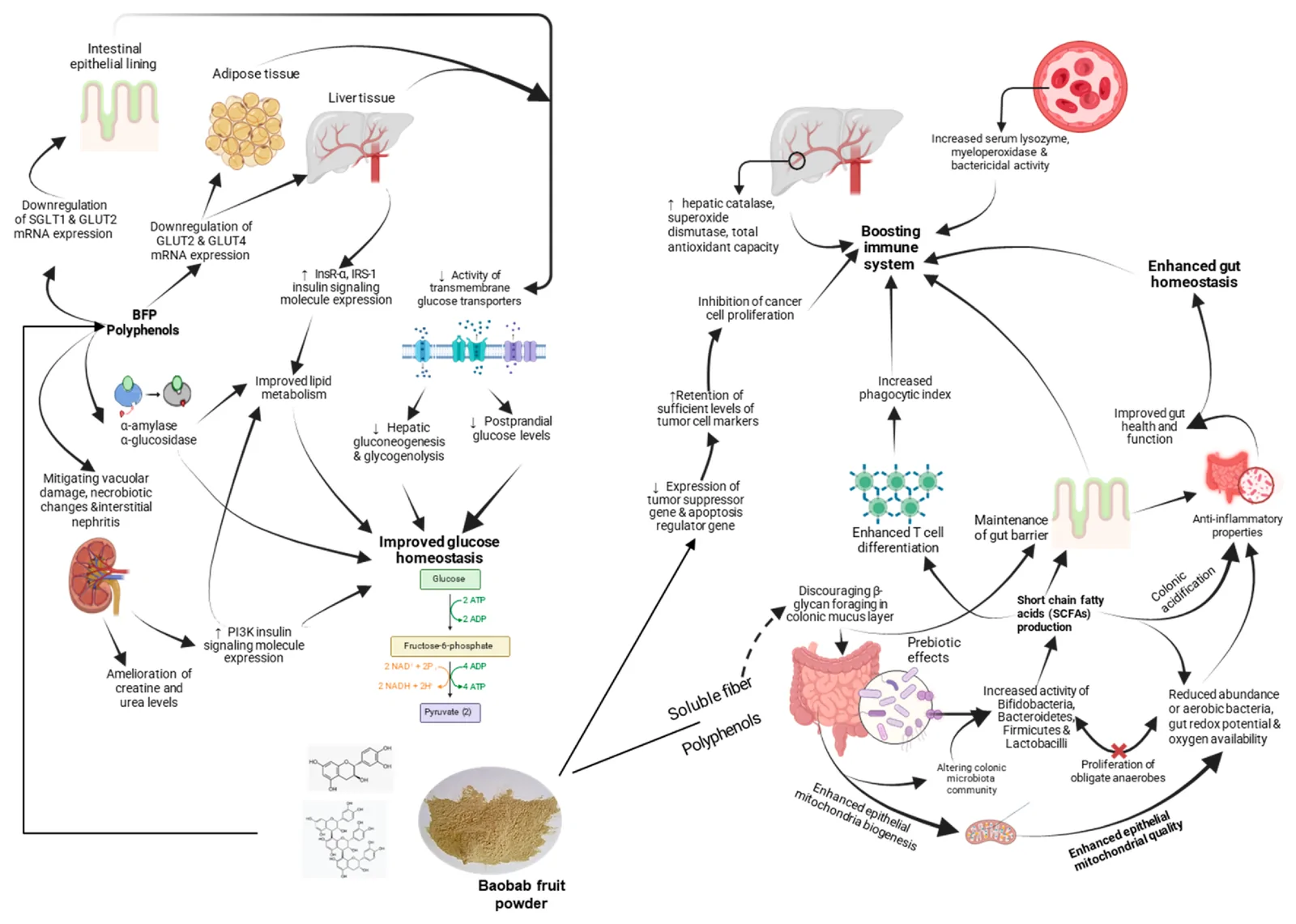
Health-promoting effects of baobab fruit powder. Solid arrows indicate pathways supported by direct BFP-specific evidence (e.g., fermentation of BFP fibers, SCFA production in in vitro models, α-amylase and α-glucosidase inhibition). Dashed arrows indicate pathways inferred from studies on other polyphenol or fiber sources (e.g., AMPK-PGC-1α-mediated mitochondrial effects, SGLT1 inhibition, GLUT2/GLUT4 modulation, NF-κB inhibition). No pathway shown has been confirmed in human intervention studies. ↑ and ↓: Indicate an increase and decrease, respectively, in the specified parameter, activity or expression level.

Dietary fiber intake has a significant influence on optimal gut function, in part through potential prebiotic effects (Figure 1). The term “prebiotic” is formally defined by the International Scientific Association for Probiotics and Prebiotics (ISAPP) as “a substrate that is selectively utilized by host microorganisms conferring a health benefit” [85]. To date, evidence for BFP falls short of this full definition, as selective utilization has been demonstrated in vitro, but a health benefit attributable to microbiota modulation has not yet been confirmed in human studies. The potential prebiotic activity of BFP is mediated through multiple complementary mechanisms. First, the low-methoxylated homogalacturonan (LMHG) content of BFP (15–20% of total fiber) provides a unique substrate for microbial fermentation. Alba et al. [39] characterized BFP polysaccharides and reported that LMHG comprises 15–20% of total fiber, with a degree of methoxylation (DM) consistently below 50% as determined by FT-IR and NMR spectroscopy. This low DM facilitates cross-linking with calcium ions, forming hydrogel structures that resist rapid degradation, thereby sustaining fermentable substrate availability throughout the colon [34]. Second, the xylogalacturonan fraction (XGA), comprising a xylose-substituted galacturonic acid backbone with a xylose:galacturonic acid ratio ranging from 1:4 to 1:8 depending on provenance as determined by monosaccharide analysis and NMR spectroscopy [44], serves as a selective substrate for specialized Bacteroides species equipped with the polysaccharide utilization loci (PUL) necessary for XGA depolymerization [86,87]. Third, the inulin and fructooligosaccharide (FOS) content of BFP (2.5–4.2% and 1.8–3.5% of total carbohydrates, respectively, as quantified by HPAEC-PAD chromatography in BFP samples from multiple provenances [42,44], specifically promotes *Bifidobacterium* proliferation in vitro, consistent with the observed increases in *Bifidobacteriaceae* abundance following BFP supplementation in fermentation models [42,43]. The prebiotic activity of these fructans has been demonstrated through in vitro fermentation studies showing selective growth promotion of *Bifidobacterium* species, with FOS exhibiting particularly strong bifidogenic effects at concentrations as low as 0.5% (*w*/*v*) [41,42]. It has been hypothesized that the synergistic effects of these three fiber fractions may explain the broader and more robust fermentative activity observed with these substrates compared with single-fiber substrates. However, this hypothesis has not been directly tested by experimentally comparing individual fiber fractions with their combination, and the proposed synergy therefore remains an inference.

While these in vitro findings are promising and mechanistically plausible, several critical gaps prevent confirmation of a prebiotic effect in humans. First, no human intervention study has directly measured changes in gut microbiota composition and function following BFP consumption. Second, no study has demonstrated a health benefit (e.g., improved gut barrier function, reduced inflammation, or enhanced immune response) that can be unequivocally attributed to BFP-induced changes in the microbiota. Natural products, including plant-derived polysaccharides and polyphenols, have been shown to remodel the gut ecosystem by modulating microbiota composition and metabolite production, thereby exerting therapeutic effects in gastrointestinal disorders [88], Third, the in vitro models used to date [42,43] do not fully recapitulate the complexity of the human gut ecosystem, including host-microbe interactions, mucosal immunity, and inter-individual variability. Therefore, we conclude that BFP demonstrates potential prebiotic activity in vitro, but confirmation of a prebiotic effect requires well-designed human intervention trials with appropriate mechanistic and health outcome endpoints.

Using an in vitro human fecal microbiota batch fermentation model, Foltz et al. [42] showed that BFP powder alters the abundance of specific microbial species in donor fecal samples. A host-dependent increase in the activity of microbiota belonging to members of the phyla *Bacteroidetes* and *Firmicutes,* as well as genera including *Bifidobacterium* and *Lactobacilli*-related species, was observed after BFP supplementation in this in vitro system. In a study involving bread formulation and simulated gastrointestinal digestion, Adedeji et al. [69] observed the potential prebiotic activity of BFP in an ex vivo model. BFP increased the viability of *Lactobacillus rhamnosus* LGG by as much as 3% during cultivation in a laboratory medium. In addition, the formulation of white-pan bread with BFP significantly increased *L. rhamnosus* viability by as much as 11% during stimulated passage through the gastrointestinal tract. Therefore, the stimulation of *Bifidobacteria* and *Lactobacilli* growth by BFP in vitro provides evidence for potential prebiotic activity in these experimental systems. However, the ISAPP definition of a prebiotic requires both selective microbial utilization and a demonstrated health benefit in the host [85]. While the in vitro evidence for selective utilization is strong, human studies demonstrating a health benefit attributable to BFP’s modulation of the gut microbiota are currently lacking.

In other donors, an increase in *Lactobacilli* was observed, and consequently, an increase in lactate, which is the main end product of pectic polysaccharide fermentation by *Lactobacilli* [42,89]. This agrees with the work of Penumutchu et al. [90], who observed reduced abundances of aerobic bacteria, gut redox potential, and oxygen availability in female mice fed a diet supplemented with purified plant fiber. As such, it has been proposed that BFP supplementation promotes selective utilization, thereby altering the bioenergetics of gut bacteria to enhance the activity of microbiota associated with dormancy, carbon fixation, and fatty acid metabolism, rather than respiratory metabolism [90]. This proposed shift in bacterial bioenergetics is extrapolated from studies using purified plant fibers and has not been directly demonstrated with BFP.

The activity of these groups of microbiota, including *Bifidobacteria* and *Lactobacilli,* is accompanied by an increase in the production of metabolites, including acetate, propionate, and butyrate (short-chain fatty acids, SCFAs), as well as lactate, which is a metabolic intermediate produced during carbohydrate fermentation and is subsequently converted to SCFAs by cross-feeding bacteria [42]. Therefore, fermentation of BFP pectic polysaccharides leads to the production of SCFAs, which exert anti-inflammatory properties, maintain intestinal barrier integrity, and promote gut motility [91]. The anti-inflammatory properties of fruit-derived polysaccharides have been demonstrated in various experimental models, with mechanisms including modulation of inflammatory cytokine production and enhancement of intestinal barrier function [92]. SCFAs also play a role in colonic acidification, which drives colonization resistance by reducing pathogen growth in the gut [86]. The therapeutic potential of plant-derived polysaccharides in metabolic diseases has been increasingly recognized, with mechanisms including modulation of gut microbiota, enhancement of SCFA production, and regulation of glucose and lipid metabolism [93]. However, evidence directly linking BFP consumption to reduced pathogen colonization or infection risk in humans is currently lacking.

Using the SHIME^®^ (Simulator of the Human Intestinal Microbial Ecosystem), an in vitro dynamic gut model that mimics the proximal and distal colon regions, Duysburgh et al. [43] investigated the influence of BFP, Arabic gum, and their combination on human gut microbiota responses, using an in vitro gut model simulator. Gut microbial activity and composition were quantitatively monitored by 16S-targeted Illumina sequencing. The results show that repeated administration of BFP, either alone or in combination with Arabic gum, significantly affected metabolic activity, diversity index, and community composition within this simulated system. Advanced analytical approaches, including 16S rRNA sequencing and metabolomics, have enabled detailed characterization of gut microbiota-metabolite interactions, providing insights into the mechanisms by which dietary fibers influence host health [94]. Specific effects of the treatment were observed for *Bifidobacteriaceae* and *Faecalibacterium prausnitzii*, which were prominent in the simulator’s distal and proximal colon. Metabolic activity also increased over long-term experimentation in the simulator, as indicated by increases in total SCFAs in the proximal and distal colonic regions [43]. Additionally, a consistent reduction in bCFA (the sum of isobutyrate, isovalerate, and isocaproate) and ammonium levels was observed in both colonic regions in this in vitro system. These markers are associated with proteolytic fermentation, and their reduction has been suggested as a potential indicator of favorable gut conditions in some studies [43]. However, direct evidence linking BFP-induced changes in these markers to reduced cancer risk or improved health outcomes in humans is currently lacking. These in vitro findings demonstrate the potential prebiotic activity of BFP in a simulated gut environment. However, confirmation of a prebiotic effect in humans, defined as selective microbial utilization with a demonstrated health benefit in the host, requires well-designed human intervention studies with appropriate gut health and clinical endpoints [85].

It is important to emphasize that all evidence to date of BFP-induced gut microbiota modulation derives from in vitro or ex vivo models. While these models provide valuable mechanistic insights, they do not fully recapitulate the complexity of the human gut ecosystem, including host-microbe interactions, mucosal immunity, inter-individual variability, and the influence of the host diet and physiology. Therefore, findings from these models should be considered preliminary and hypothesis-generating rather than confirmatory of effects in humans. Well-designed human intervention studies with appropriate microbiota and health outcome endpoints are urgently needed to translate these promising in vitro findings to clinical applications.

#### 4.1.2. Effect of Baobab Fruit Powder Polyphenols on Gut Function

Based on its function, the gut is continuously exposed to pathogens and toxins, which affects its viability. Cumulative exposure can trigger cell damage and apoptosis, thereby requiring high epithelial cell turnover to maintain intestinal integrity [95]. This imposes high energy demands on the intestines, which, in turn, affect intestinal epithelial function, the performance of which requires high mitochondrial quality. Research has highlighted the bidirectional interactions between the mitochondria of intestinal epithelial cells, mucosal cells, immune cells, and gut microflora [81,82]. Mitochondrial dysfunction in intestinal epithelial cells is directly linked to gut dysbiosis, highlighting mitochondria’s potential as therapeutic targets for treating gut microbiota disorders.

Certain polyphenols identified in BFP, including epicatechin gallate (ECG), epigallocatechin gallate (EGCG), and procyanidin dimers [16,18,52], are structurally similar to compounds with established bioactivities in other contexts. However, it is important to distinguish between mechanisms that have been directly demonstrated using BFP-specific preparations and those that are extrapolated from the general polyphenol literature based on the presence of structurally similar compounds in BFP [96,97]. BFP-specific evidence for mitochondrial modulation is currently limited. No study has directly tracked BFP-derived polyphenols through the gastrointestinal tract and demonstrated their specific effects on intestinal epithelial mitochondrial function in vivo. The proposed mechanism is extrapolated from studies on structurally similar polyphenols from other dietary sources (e.g., green tea catechins, grape proanthocyanidins), which activate AMP-activated protein kinase (AMPK) via phosphorylation at Thr172, leading to phosphorylation and activation of peroxisome proliferator-activated receptor gamma coactivator 1-alpha (PGC-1α), a master regulator of mitochondrial biogenesis [79,82]. It should be noted that while increased oxygen consumption is consistent with enhanced mitochondrial activity, it is not a definitive measure of improved mitochondrial function. Mitochondrial health encompasses multiple parameters, including membrane potential, ATP production efficiency, reactive oxygen species generation, and mitochondrial biogenesis. Studies directly measuring these parameters in BFP-treated models are currently lacking, and the proposed mitochondrial mechanism therefore remains largely inferential.

This pathway enhances mitochondrial oxidative phosphorylation, thereby increasing oxygen consumption in epithelial cells as a proxy for mitochondrial activity and promoting physiological hypoxia in the colonic luminal cavity [83]. In studies using non-baobab polyphenol sources, these hypoxic conditions have been reported to selectively discourage facultative anaerobic *Enterobacteriaceae* while favoring obligate anaerobes such as *Bifidobacterium* and *Lactobacillus* [98]. This downstream effect has not been demonstrated with BFP-specific preparations and remains an extrapolation. However, increased oxygen consumption alone does not independently demonstrate improved mitochondrial function; it reflects enhanced electron transport chain activity but does not measure mitochondrial biogenesis, membrane potential, or ATP production efficiency. Direct measures of mitochondrial function, including mitochondrial DNA copy number, citrate synthase activity, or ATP:ADP ratios, are needed to confirm improved mitochondrial health [95,97]. While this mechanistic chain is biologically plausible and consistent with the known bioactivities of BFP’s constituent polyphenols, the complete pathway from BFP polyphenol consumption through to in vivo mitochondrial effects and subsequent microbiota modulation has not been directly demonstrated in BFP-specific studies (Table 7).

A critical question is whether BFP contains these polyphenols at concentrations sufficient to exert the proposed effects in vivo. Based on available quantitative data, the estimated concentrations of key polyphenols in BFP are as follows: procyanidin B2 (0.5–2.0 mg/g), catechin/epicatechin (major components by NMR, estimated 1–5 mg/g), chlorogenic acid (0.5–1.5 mg/g), and ferulic acid (0.2–0.8 mg/g) [17,18,50]. When considering typical BFP consumption levels of 10–20 g per serving, the estimated intake of procyanidin B2 would be 5–40 mg per serving. By comparison, the IC_50_ values for AMPK activation by related polyphenols in vitro are typically in the micromolar range (approximately 10–100 μM), which corresponds to 5–50 μg/mL in cell culture [97,99]. However, these in vitro effective concentrations do not account for the bioavailability, metabolism, and tissue distribution of BFP polyphenols in humans. To date, no pharmacokinetic studies have been conducted to determine the systemic concentrations of BFP polyphenols following oral consumption. Therefore, while the polyphenol content of BFP is qualitatively consistent with the proposed mechanisms, quantitative confirmation of physiologically meaningful concentrations at target tissues remains an open question.

#### 4.1.3. Synbiotic Potential of Baobab Fruit Pulp

The current surge in the prevalence of non-communicable diseases, including metabolic syndrome, has led to increased interest in probiotic and prebiotic interventions to improve gut health [80,100]. Together, probiotics and prebiotics can provide synergistic benefits for the host compared with their individual effects [97]. This necessitates consideration of the synbiotic concept, defined as “a mixture comprising live microorganisms and substrate(s) selectively utilized by host microorganisms that confer a health benefit on the host” [97,98]. According to this definition, demonstration of a synbiotic effect requires both selective substrate utilization by the probiotic organism and a documented health benefit in the host, not merely improved probiotic survival or growth during product fermentation. Fowoyo et al. [41] confirmed the presence of inulin and fructooligosaccharides in BFP and demonstrated successful development of non-dairy synbiotic beverages using BFP fermented with *Limosilactobacillus fermentum*. Probiotic viability remained at 10^7^–10^8^ CFU/mL over three weeks of storage at both room and refrigeration temperatures, supporting BFP’s utility as a prebiotic substrate in functional food applications. Furthermore, the BFP phytochemical profile, consisting of flavonoids, phenolic acids, and organic acids, contributes to its nutritional and medicinal properties, which are essential for optimal gut function [16,17,51]. Fowoyo et al. [41] demonstrated the potential prebiotic activity and antimicrobial effects of BFP in vitro, which is in agreement with the findings of Foltz et al. [42] and Thompson et al. [99]. Therefore, BFP provides an opportunity for targeted remodeling of the human gut microbiome by modulating microbial composition and promoting the growth of beneficial bacteria such as *Bifidobacteriaceae*, *Lactobacilli*, and *Faecalibacterium prausnitzii*, as well as enhancing the production of short-chain fatty acids (SCFAs). However, these findings are based on in vitro models and do not constitute evidence for a synbiotic effect in humans.

The addition of BFP to fermented dairy products has been explored as a strategy to support probiotic viability and improve food safety. Mpofu et al. [71] demonstrated that BFP addition promoted the growth of *Lactobacillus rhamnosus* yoba in mutandabota, a traditional fermented milk product from Zimbabwe. The acidic pH (3.5 ± 0.1) of the product, attributed to BFP organic acids, contributed to the inactivation of pathogenic bacteria, including *Listeria monocytogenes*, *Escherichia coli*, and *Salmonella* spp., thereby improving microbiological safety [70,71]. While these findings demonstrate BFP’s potential as a prebiotic substrate in fermented foods and its utility in food safety applications, they do not provide direct evidence for modulation of the human gut microbiota or synbiotic health effects in consumers. The relevance of these findings to the mechanistic scope of this review lies in their demonstration of BFP’s fermentable fiber content and acidifying properties, which are consistent with the in vitro prebiotic activity discussed in Section 4.1.1. Overall, BFP shows potential for use in the production of functional foods with symbiotic potential. However, confirmation of a synbiotic effect requires human intervention studies demonstrating that the combination of BFP and specific probiotic strains produces a health benefit greater than that of the probiotic or prebiotic alone. To date, such studies have not been conducted.

### 4.2. Potential Immunomodulatory Effects of Baobab Fruit Pulp

The current consumer approach to prevention of over-cure has attracted increasing interest in foods with proposed immunomodulatory properties [101]. Therefore, the nutrition industry is interested in functional ingredients with biologically active compounds that impart immune-modulating, antioxidant, anti-inflammatory, antitoxic, and ergogenic effects on food. The available evidence for the immunomodulatory effects of BFP is predominantly derived from fish, rodent, and cell-culture models. No human intervention studies have examined immune outcomes following BFP consumption, and no demonstrated immune benefit in humans has been reported. The interplay between gut microbiota, intestinal barrier function, and immune regulation has been extensively studied in the context of metabolic diseases. For example, plant-derived polysaccharides have been shown to improve intestinal barrier function and modulate immune responses through microbiota-dependent mechanisms [102]. Based on ethnobotanical evidence, the proposed immunostimulatory effects of baobab fruit have also been investigated in animal and cell-culture models. Hendam et al. [72] used a fish model to evaluate the potential of dietary BFP powder as a growth-stimulant, antioxidant, and immunomodulatory agent in aquaculture. Supplementation was associated with increased levels of serum immune biomarkers, including lysozyme, myeloperoxidase, and bactericidal activities in the fish model [72]. Increased expression of growth-related genes was also observed, as well as a dose-dependent increase in disease resistance in this model. Furthermore, groups administered BFP showed an increase in hepatic catalase, superoxide dismutase, and total antioxidant capacity, coupled with a decrease in malondialdehyde concentrations. This highlights the ability of BFP to increase hepatic antioxidant capacity, which is in agreement with earlier studies by [74]. The hepatoprotective effects were attributed to the activities of BFP compounds, including triterpenoids, β-sitosterol, β-amyrin palmitate, α-amyrin, and ursolic acid [72,74]. While these findings suggest immunostimulatory activity in fish, they do not establish that such increases would be beneficial or safe in humans.

Badu et al. [103] confirmed the anti-inflammatory activity of BFP extracts using a carrageenan-induced foot edema model in chicks, with an ED_50_ value of 282.12 mg/kg body weight. Acute toxicity evaluation revealed no mortality at the highest administered dose of 5000 mg/kg BW (LD_50_ > 5000 mg/kg), and 28-day sub-acute toxicity studies showed no adverse effects on hematological or biochemical parameters, with histopathological analysis confirming the absence of kidney or liver damage. These findings provide important safety data for BFP consumption. In another study, the effect of BFP on Delayed Type Hypersensitivity (DTH) response in Wistar rats treated with Sheep Red Blood Cells (SRBC) was investigated [68]. Delayed hypersensitivity is an exaggerated immune response mediated by antigen-specific T cells that leads to tissue damage. After five days of oral administration, the study observed that BFP extracts were associated with increased delayed-type hypersensitivity reactions. Administration of BFP extracts to immunosuppressed rats also led to an increased SRBC-induced antibody titer and an increase in the phagocytic index [68]. However, an enhanced DTH response may indicate excessive inflammatory activation rather than a beneficial immunostimulatory effect [30,104]. In the absence of functional immune outcomes (e.g., reduced infection incidence, improved vaccine response) or safety assessments (e.g., autoimmune markers, inflammatory cytokine profiles), the biological relevance and potential adverse implications of these findings remain unclear.

It is proposed, based on the established effects of SCFAs in the broader dietary-fiber literature, that the proposed immunomodulatory properties of BFP may arise from the effect of gut microbiota metabolites, particularly short-chain fatty acids (SCFAs), in stimulating T cell differentiation in animal models through specific G protein-coupled receptors [104]. However, this mechanistic chain, from BFP consumption to SCFA production to T-cell differentiation, has not been directly demonstrated using BFP-specific preparations. Furthermore, the link between BFP-induced SCFA production and T-cell differentiation remains inferential; no study has directly tracked BFP-derived SCFAs to specific T-cell subsets in vivo. As such, BFP has demonstrated immunomodulatory activity in animal models and potential as an immunomodulatory agent, given its ability to promote the fermentative activity of commensal microbes in vitro that produce biologically active metabolites. However, human translation of these findings has not been established. Foltz et al. [42] and Duysburgh et al. [43] demonstrated that BFP fermentation in in vitro gut models increases SCFA production (acetate, propionate, butyrate) and alters microbial composition. However, no study has directly demonstrated that BFP-fermentation-derived SCFAs specifically promote Treg differentiation or modulate immune function through GPR43/GPR109A signaling in BFP-treated subjects. Acetate, propionate, and butyrate activate GPR43 and GPR109A on regulatory T cells (Tregs), promoting the differentiation of naïve CD4^+^ T cells into FoxP3^+^ Tregs via histone deacetylase (HDAC) inhibition and enhanced histone acetylation at the FoxP3 locus [105,106]. This signaling cascade, mediated by modulation of the PI3K/Akt/mTOR pathway, enhances Treg immunosuppressive function and reduces the Th17/Treg ratio, thereby mitigating chronic inflammation [107]. This mechanistic chain is biologically plausible and consistent with the known bioactivity of SCFAs in other contexts [105,106,107], but the specific demonstration that BFP-derived SCFAs activate these pathways in vivo has not been established. This represents a significant evidence gap that should be prioritized in future research. Additionally, butyrate promotes the expression of antimicrobial peptides (defensins and cathelicidins) in intestinal epithelial cells through activation of the GPR109A-AMPK pathway [108].

It has been hypothesized, based on general dietary-fiber studies, that the pectic polysaccharides in BFP may contribute to maintaining the integrity of the colonic mucus barrier. The colonic mucus layer serves as a dynamic barrier critical to pathogen invasion. It is composed of the mucin-2 glycoprotein, with 80% of the mucin biomass consisting mostly of *O*-linked glycans [86]. When a sustained deficiency of dietary fiber occurs, gut microbiota, such as *Bacteroides thetaiotaomicron*, are capable of foraging for glycans from the host mucus layer, thereby depleting it [87]. Desai et al. [86] showed that a deficiency of dietary fiber promotes erosion of the colonic mucus barrier, as gut microbiota digest mucus glycoproteins as a nutrient source. The resulting intestinal barrier dysfunction creates easier access to mucosal pathogens, including *Citrobacter rodentium*. While this mechanism is biologically plausible and consistent with the known fermentability of BFP fibers [42,43], it is important to emphasize that the specific effect of BFP on mucus-barrier integrity has not been directly demonstrated. The specific effect of BFP on mucus-barrier integrity has not been directly demonstrated. The evidence for this proposed mechanism is extrapolated from studies using other fiber sources [86,87] and requires confirmation using BFP-specific preparations in appropriate models.

In addition to the immunomodulatory effects discussed above, BFP extracts have been investigated for antiproliferative and antitumor activity in cell lines and animal models. These findings are distinct from immunomodulatory effects and should be interpreted separately, with appropriate attention to the limitations of the experimental models used. El-Masry et al. [75] investigated the antiproliferative activity of BFP extract in human colon cancer (HCT116), human breast cancer (MCF-7), and human ovarian cancer (OVCAR-3 and OVCAR-4) cell lines. Methanolic extracts of fruit fibers demonstrated antiproliferative activity against HCT116 and MCF-7 cells and altered gene expression in these cell lines in vitro. The study further analyzed gene expression profiles in HCT116 and MCF-7 cell lines treated with methanolic fiber extracts. The significantly enriched pathways were those for cell cycle/cell proliferation and programmed cell death/apoptosis [75]. These findings demonstrate that BFP extracts can modulate cancer-relevant pathways in cultured cells, but they do not establish efficacy in humans. Similarly, Elsaid [76] observed that BFP extracts were associated with reduced tumor growth in a mouse model of Ehrlich ascites carcinoma (EAC) cells, with concomitant modulation of redox state and tumor marker levels. In their experiment, the sera of female BALB/C mice administered BFP extract retained sufficient activity levels of tumor markers, including α-l-fucosidase and arginase. This is significant because serum levels of α-l-fucosidase are reliable biomarkers for detecting hepatocellular carcinoma, with sensitivity and specificity of 85% and 91%, respectively [109]. Furthermore, BFP extracts were associated with restoration of *p53* tumor suppressor gene and the *Bcl-2* apoptosis regulator gene expression toward normal levels in the EAC mouse model [109]. This finding is biologically plausible as p53 is a tumor suppressor; its restoration (rather than attenuation) would be consistent with reduced tumor growth. The original study reported that baobab extracts had an ‘anti-apoptosis role by restoring the p53 and Bcl-2 gene expression’ [109]. Therefore, the ability of BFP extracts to regulate gene expression and restore serum redox balance in animal models highlights their potential as a candidate for further investigation in cancer research. However, these findings are derived from cell lines and animal models; their relevance to human cancer prevention or treatment has not been established, and such claims should not be extrapolated to clinical outcomes without direct evidence.

### 4.3. Effects of BFP on Postprandial Glycemic Response and Glucose Metabolism

The evidence for BFP-mediated glycemic regulation spans in vitro enzyme inhibition studies, animal models, and limited human intervention trials. This section presents findings by study type, with explicit attention to the translational relevance of each level of evidence. Salih et al. [110] showed that BFP extracts significantly reduced blood sugar levels in diabetic rats, thereby providing therapeutic effects in managing diabetes-associated inflammation. This was consistent with earlier studies by Coe et al. [77], who showed that baobab extracts reduced bread starch breakdown and sugar release during the duodenal phase of digestion. The observed glycemic response was attributed to the BFP polyphenol fraction and its soluble fiber, acting through multiple molecular pathways. First, BFP polyphenols interfere with starch breakdown by inhibiting the activities of α-amylase and α-glucosidase through direct binding to enzyme active sites. Catechin and quercetin interact with residues R407, R197, R407, and F282 on the α-glucosidase active site, while catechin, quercetin, and rutin interact with the K200, E233, D300, W151, A198, and L162 residues at the α-amylase via hydrogen bond and electrostatic interactions, causing competitive inhibition [57]. Second, it has been hypothesized that BFP galloylated polyphenols (ECG and EGCG) may modulate intestinal glucose absorption by competitively inhibiting the sodium-dependent glucose transporter 1 (SGLT1). In studies using green tea polyphenols, ECG and EGCG bind to the glucose-binding site of SGLT1, reducing the transmembrane transport of glucose from the intestinal lumen into enterocytes [59,111]. This mechanism has not been directly tested with BFP and is inferred from studies on other polyphenol sources.

Third, preliminary evidence suggests that BFP polyphenols may modulate the activity of dipeptidyl peptidase-IV (DPP-IV), the enzyme responsible for degrading incretin hormones (GLP-1 and GIP). Cicolari et al. [57] reported DPP-IV inhibition by BFP extracts with an IC_50_ range of 45–80 μg/mL in vitro, providing the only BFP-specific evidence for this mechanism to date. However, this finding requires replication and extension to in vivo models, and the clinical relevance of BEF-mediated DPP-IV inhibition remains unestablished. This triple mechanism enzyme inhibition, transporter modulation, and incretin preservation has been proposed as a molecular framework for understanding BFP’s glycemic effects. However, only the enzyme-inhibition component has been directly demonstrated using BFP-specific preparations; the transporter modulation and incretin-preservation components are inferred from studies of structurally similar polyphenols from other sources and require direct confirmation with BFP. Recent clinical evidence supports the glycemic benefits of BFP, with Rita et al. [78] demonstrating a significant reduction in postprandial glycemic response in healthy adults (n = 31; mean reduction in incremental area under the curve: 18.7%, *p* < 0.05). However, the clinical significance of this finding requires careful interpretation, as the study population consisted of normoglycemic individuals, and the reduction, while statistically significant, may not translate to clinically meaningful outcomes in diabetic populations. Coe et al. [77] demonstrated dose-dependent reductions in glycemic response (low dose: 12.5%; high dose: 23.8%) after consuming white bread with BFP extract, suggesting a concentration-dependent effect. These human findings are mechanistically supported by the in vitro evidence presented in Section 4.3.1. As summarized in Table 8, the inhibition of α-amylase and α-glucosidase has been directly demonstrated using BFP-specific preparations, while the proposed involvement of SGLT1, GLUT2/GLUT4, and DPP-IV remains inferred from studies on structurally similar compounds from other sources. The relative contribution of each mechanism to the observed glycemic effects in humans has not been established.

Rita et al. [78] stated that baobab polyphenols may exert a regulatory effect on enteric glucose absorption. Glucose transport within the small intestine and other tissues is facilitated by transmembrane carrier proteins, including glucose transporter 2 (GLUT2) and sodium-glucose transporter 1 (SGLT1). Type II diabetes is associated with upregulated SGLT1 and GLUT2 mRNA and protein levels in the intestinal epithelium, leading to elevated postprandial glucose levels [112]. Galloylated polyphenols, including epicatechin gallate (ECG) and epigallocatechin gallate (EGCG), inhibit the activity of the sodium-dependent glucose transporter SGLT1 in the intestinal tract [59]. Alternatively, activation of glucose utilization in peripheral tissues, such as the liver, adipose tissue, and muscles, can play an important role in blood glucose homeostasis. Polyphenols can modulate the expression of GLUT2 and GLUT4 mRNA in the liver and adipose tissue, respectively [58]. This alters hepatic gluconeogenesis and glycogenolysis in these tissues, contributing to glucose homeostasis in diabetic subjects [58].

Sulaiman et al. [8] investigated the effects of BFP in a rat model of metabolic syndrome induced by a high-sugar/high-fat (HS/HFD) diet. Supplementation with BFP was associated with dose-dependent reductions in serum creatinine and urea levels in this rat model, suggesting potential renoprotective effects that require confirmation in human studies. Changes in the Atherogenic Index of Plasma (AIP) were observed in BFP-treated rats, but these findings from an animal model do not establish cardiovascular risk reduction in humans. BFP administration was associated with weight loss in this rat model of diet-induced obesity. BFP supplementation was associated with improvements in lipid profile, postprandial plasma glucose, and liver function markers in this animal model. The observed changes in lipid profiles in this model were attributed to inhibition of key enzymes, including α-amylase, α-glucosidase, angiotensin-converting enzyme, 3-hydroxy-3-methyl-glutaryl-coenzyme A reductase, and pancreatic lipase [61]. The administration of polyphenols can also increase the expression of insulin signaling molecules, including the insulin receptor α subunit (InsR-α), insulin-receptor substrate-1(IRS-1), and phosphatidylinositol 3-kinase (PI3K) in kidney tissue in animal models [96]. Supplementation with BFP was associated with reduced histological damage in liver and kidney tissues in this rat model. Similarly, Baky et al. [50] observed improved kidney histology after BFP treatment compared with diabetic rat subjects, which exhibited vacuolar degeneration, necrobiotic changes, and interstitial nephritis. These histological observations in rats suggest potential renoprotective effects, but do not establish therapeutic efficacy in humans.

#### 4.3.1. In Vitro and Molecular Evidence

Mechanistic studies on BFP’s glycemic effects can be categorized into two groups: (1) mechanisms with direct BFP-specific evidence, and (2) mechanisms inferred from the presence of structurally similar compounds in BFP. Table 8 summarizes the evidence status for each proposed mechanism. Cicolari et al. [57] demonstrated that hydromethanolic extracts from BFP inhibit α-amylase (IC_50_ 9.97 mg/mL) and α-glucosidase (IC_50_ 0.64 mg/mL) in vitro. Molecular docking studies using BFP-identified compounds (catechin, quercetin, rutin) revealed specific binding interactions: catechin and quercetin interact with residues R407, R197, R407, and F282 on the α-glucosidase active site, while catechin, quercetin, and rutin bind to K200, E233, D300, W151, A198, and L162 residues on α-amylase via hydrogen bonds and electrostatic interactions [57]. Mohammed et al. [17] confirmed that chlorogenic and ferulic acids from BFP exhibit similar inhibitory effects through hydrogen bonding with catalytic residues. These findings provide a mechanistic basis for the observed glycemic effects in animal and human studies discussed in Section 4.3.2 and Section 4.3.3. Inferred mechanisms (transporter modulation): It is hypothesized, based on the presence of galloylated polyphenols (ECG, EGCG) in BFP [16,18,52] and their known bioactivities in other contexts, that BFP polyphenols may influence glycemic control through modulation of glucose transporters. Kobayashi et al. [59] demonstrated that ECG and EGCG from green tea competitively inhibit intestinal SGLT1, while other polyphenols have been shown to modulate hepatic GLUT2 and adipose GLUT4 expression through AMPK-PPARγ signaling [58]. However, no study has directly tested BFP extracts or BFP-isolated compounds for SGLT1 inhibition, GLUT2/GLUT4 modulation, or DPP-IV inhibition. These proposed mechanisms are therefore inferred from the presence of structurally similar compounds in BFP and require direct confirmation using BFP-specific preparations.

#### 4.3.2. Animal Studies

Several rodent studies have investigated the glycemic effects of BFP in diabetic models. Salih et al. [110] showed that BFP extracts significantly reduced blood sugar levels in diabetic rats, with associated reductions in TNF-α, IL-6, and IL-10 levels, suggesting anti-inflammatory effects. Baky et al. [50] observed hypoglycemic effects in alloxan-induced diabetic rats treated with BFP at 150 or 300 mg/kg, along with restoration of kidney histological structure and reductions in serum ALP, ALT, and AST levels. Suliman et al. [8] demonstrated that BFP supplementation in a high-sugar/high-fat diet-induced metabolic syndrome model in Wistar rats produced dose-dependent reductions in blood glucose, improved lipid profiles, and restored organ histology. These animal studies provide proof of concept that BFP can influence glycemic parameters in diabetic models. However, findings from rodent models do not guarantee translation to humans, and the doses used (150–800 mg/kg) may not be directly comparable to typical human consumption levels. The gut-kidney axis has emerged as a critical pathway linking gut microbiota dysbiosis to chronic kidney disease, with gut microbiome-targeted therapeutics showing promise in preclinical models [100,113].

#### 4.3.3. Human Intervention Studies

Limited human evidence exists for BFP’s glycemic effects, all conducted in healthy normoglycemic adults. Coe et al. [77] demonstrated dose-dependent reductions in glycemic response (low dose: 12.5%; high dose: 23.8%) following white bread consumption with BFP extract in healthy female participants (*n* = 9), suggesting a concentration-dependent effect. Rita et al. [78] reported a significant reduction in postprandial glycemic incremental area under the curve (18.7% reduction, *p* < 0.05) in healthy adults (*n* = 31) following co-administration of BFP with glucose. These human findings are mechanistically supported by the in vitro enzyme inhibition data presented in Section 4.3.1. While these findings demonstrate that BFP can attenuate postprandial glucose excursions in healthy individuals, they should not be interpreted as evidence that BFP prevents or manages diabetes.

#### 4.3.4. From In Vitro Enzyme Inhibition to Human Glycemic Response: Translation and Limitations

The translation of in vitro enzyme inhibition findings to clinically meaningful glycemic effects in humans requires careful consideration of several factors. While BFP extracts inhibit α-amylase and α-glucosidase in vitro with IC_50_ values of 9.97 mg/mL and 0.64 mg/mL, respectively [17,57], these assays do not account for the complex physiological environment of the human gastrointestinal tract, including gastric pH, enzyme concentrations, food matrix effects, and intestinal transit time. Furthermore, the concentrations of BFP polyphenols reaching the small intestine after oral consumption are likely lower than those tested in vitro due to limited bioavailability and metabolism. Coe et al. [60] reported dose-dependent reductions in postprandial glycemic response (12.5% and 23.8% for low and high doses, respectively) in healthy female participants (*n* = 9). Rita et al. [78] observed a significant reduction in postprandial glycemic incremental area under the curve (18.7%, *p* < 0.05) in healthy adults (*n* = 31). However, neither study measured insulin levels, and no studies have been conducted in diabetic or prediabetic populations. The effects on insulin secretion, insulin sensitivity, or long-term glycemic control (HbA1c) remain unknown.

The high soluble fiber content of BFP (45.8–56.62 g/100 g dw, primarily as pectic polysaccharides and inulin/FOS) [40,42,45] can: (i) increase digesta viscosity, slowing gastric emptying and reducing the rate of glucose absorption [91]; (ii) form gel-like structures in the small intestine, physically entrapping starch and delaying enzymatic hydrolysis [39]; and (iii) modulate the gut microbiota, producing SCFAs that may influence glucose metabolism through gut–brain signaling and incretin hormone secretion [104,114]. These fiber-mediated effects are not captured by in vitro enzyme inhibition assays and may contribute to the observed glycemic effects in human studies. The relative contribution of enzyme inhibition versus fiber-mediated effects remains unclear. No study has systematically compared the glycemic effects of whole BFP (containing both polyphenols and fiber) with fiber-depleted BFP or isolated polyphenol fractions. Without such comparative studies, it is not possible to attribute the observed glycemic effects specifically to polyphenol-mediated enzyme inhibition, fiber-mediated viscosity effects, or synergistic interactions between components.

A critical limitation in interpreting the proposed molecular mechanisms of BFP polyphenols is the absence of pharmacokinetic data. The in vitro IC_50_ values reported for α-amylase and α-glucosidase inhibition (9.97 mg/mL and 0.64 mg/mL, respectively) and for AMPK activation (10–100 µM) cannot be directly extrapolated to human target tissue exposure. Bridging this gap requires quantitative data on several physiological processes that have not been characterized for BFP polyphenols. First, first-pass metabolism in enterocytes and the liver substantially reduces systemic concentrations of dietary polyphenols, with much of the ingested dose converted to glucuronidated, sulfated, or methylated metabolites with altered bioactivity [115]. Second, colonic microbial degradation transforms unabsorbed polyphenols into low-molecular-weight phenolic acids, which are the predominant circulating metabolites, though their enzyme-inhibitory potency may differ from that of the parent compounds [116]. Third, plasma protein binding determines the free fraction available to interact with target tissues, and this parameter has not been measured for BFP-derived polyphenols. Fourth, the food matrix in which BFP is consumed may further modulate polyphenol release, absorption, and metabolism [117]. Given these unresolved factors, the relationship between BFP polyphenol intake and target tissue exposure remains undetermined. Consequently, the proposed mechanisms of enzyme inhibition, SGLT1 modulation, GLUT2/GLUT4 regulation, and DPP-IV inhibition remain plausible but unconfirmed in humans, and the glycemic effects observed in human studies cannot be definitively attributed to polyphenol-mediated mechanisms rather than to fiber-mediated effects. Future studies should prioritize pharmacokinetic profiling of BFP polyphenols, including quantification of circulating parent compounds and metabolites, to establish whether physiologically relevant concentrations are achieved at target tissues.

### 4.4. Effects of BFP on Iron Bioaccessibility, Bioavailability, and Iron Status: Evidence from In Vitro and Human Studies

The sub-Saharan region is one of the most nutritionally insecure areas worldwide. The most nutritionally vulnerable groups include infants, young children, as well as pregnant and lactating mothers [118]. One of the key interventions to improve maternal and child nutrition is strengthening complementary feeding practices that incorporate indigenous and traditional foods. Throughout many communities in sub-Saharan Africa, indigenous fruits are valued as important drivers of nutritional security. This is particularly true for many resource-scarce communities that use these fruits for food fortification [119]. Within these communities, food-to-food fortification is promoted and practiced based on the nutritional value and acceptability of the food ingredients [65,119]. Baobab is a food ingredient used in food-to-food fortification to combat micronutrient deficiency.

It is important to distinguish between iron bioaccessibility and iron bioavailability, as these terms are not interchangeable and refer to different physiological processes. Iron bioaccessibility refers to the fraction of iron released from the food matrix during gastrointestinal digestion and available for intestinal uptake. It is typically measured using in vitro simulated digestion models (e.g., the INFOGEST protocol) that mimic gastric and intestinal conditions and do not account for intestinal uptake, transport, or systemic regulation. Iron bioavailability, by contrast, refers to the fraction of ingested iron that is absorbed and utilized or stored by the body. It can be measured in humans using stable isotope techniques or serum ferritin responses. In vitro Caco-2 cell models approximate intestinal uptake but do not measure true bioavailability because they do not recapitulate in vivo conditions, including hepcidin regulation, inflammatory status, and body iron stores. Iron uptake refers to the transfer of iron from the intestinal lumen into enterocytes, measured in Caco-2 cell models by quantifying cellular iron accumulation after exposure to digests. Iron absorption refers to the net transfer of iron from the intestinal lumen into the systemic circulation; in humans, this is measured by changes in serum iron levels or stable-isotope enrichment. Iron status is a systemic measure of body iron stores, typically assessed in humans by serum ferritin, hemoglobin, transferrin saturation, or soluble transferrin receptor (sTfR), and reflects long-term iron balance rather than acute absorption from a single meal. These distinctions are critical for interpreting the evidence presented below, and the terms are used consistently throughout this review in accordance with the definitions provided in Section 2.3. Table 9 summarizes the distinction of iron-related findings by experimental model and parameter measured.

Despite promising in vitro evidence for BFP-mediated enhancement of iron bioaccessibility [60,64,65,67] and bioavailability in the Caco-2 cell model, translation into improved iron status in human populations remains equivocal. The sole human intervention trial to date, conducted by Evang et al. [79] with Kenyan schoolchildren (*n* = 58), reported increased hemoglobin but failed to demonstrate significant improvements in serum ferritin or sTfR after 12 weeks of BFP supplementation. As discussed below, the interpretation of this result is constrained by the absence of direct absorption measurement, the possible contribution of co-nutrients to hemoglobin synthesis, and the lack of inflammation-adjusted ferritin data. Several factors may explain this discrepancy between in vitro and clinical findings: (i) the inhibitory effects of concomitant dietary factors (phytates, other polyphenols) may outweigh BFP’s enhancing effects in real-world dietary contexts; (ii) the intervention duration (12 weeks) may be insufficient for measurable changes in iron status; (iii) the baseline iron status of the study population (mild anemia, hemoglobin < 12.2 g/dL) may require more intensive intervention; (iv) differential bioavailability between BFP samples of different provenance (variation in ascorbic acid content ranges from 163.8 to 288.9 mg/100 g [47]; and (v) the intervention’s failure to align with a meal known to strongly inhibit iron absorption (e.g., high-phytate meals) may have limited the observable enhancing effect of ascorbic acid from BFP [99].

A further consideration is that the observed increase in hemoglobin in the absence of changes in ferritin or sTfR may not reflect improved iron absorption at all. Hemoglobin synthesis requires multiple nutrients in addition to iron, including vitamin C, folate, vitamin B12, and copper. BFP contains ascorbic acid (163.8–288.9 mg/100 g dw) [42,44] and copper (0.53–1.6 mg/100 g dw) [34,42], and the trial delivered BFP within a school meal, which may also have provided folate and other hematopoietic nutrients. Consequently, the hemoglobin response observed by Evang et al. [75] could plausibly reflect the combined effect of several nutrients in BFP and the accompanying meal rather than enhanced iron absorption specifically. Additionally, the interpretation of ferritin as an outcome measure in this trial is limited by its acute-phase nature, which can be elevated by subclinical infection or inflammation independently of iron status. The study did not report C-reactive protein (CRP) or alpha-1-acid glycoprotein (AGP), which are required to adjust ferritin values for inflammation in apparently healthy populations [120]. In schoolchildren in malaria-endemic and infection-prone settings, the prevalence of subclinical inflammation can be substantial, and unadjusted ferritin values may therefore have masked a true change in iron stores. The absence of inflammation-adjusted ferritin data, together with the absence of a direct measurement of iron absorption, means that the trial cannot distinguish between a genuine absence of effect on iron absorption and a methodological limitation in outcome assessment. These considerations should be taken into account when interpreting the equivocal human evidence for BFP and iron status, and future trials should measure iron absorption directly (e.g., stable isotope methods), report inflammation-adjusted ferritin (using CRP and AGP), and account for co-nutrient effects on hemoglobin synthesis.

The mechanistic basis for BFP’s iron-enhancing effects has been proposed to arise from the known activities of its constituent organic acids. Ascorbic acid (present at 175–289 mg/100 g) [45] reduces ferric iron (Fe^3+^) to the more bioavailable ferrous form (Fe^2+^), while citric and malic acids chelate iron, maintaining solubility at physiological pH [66,114]. These effects are well documented for isolated organic acids, but their net effect in the context of whole BFP and complex food matrices remains to be fully established. However, the magnitude of this effect in vivo is likely attenuated by concomitant dietary inhibitors and individual variability in iron absorption capacity. It is important to reiterate that the in vitro studies cited in this section measure iron bioaccessibility, not bioavailability. Enhancement of bioaccessibility in simulated digestion models indicates that more iron is released from the food matrix and is potentially available for uptake, but it does not demonstrate that this iron is absorbed, utilized, or stored in the body. The Caco-2 cell model provides an intermediate measure of intestinal uptake but does not account for systemic regulation by hepcidin, inflammatory status, or body iron stores. Only direct measurement of iron absorption (e.g., stable isotope techniques) or of iron status biomarkers (ferritin, sTfR, hemoglobin) can establish that BFP improves iron bioavailability or iron status in humans.

The net effect of BFP on iron bioavailability reflects a balance between favorable factors (ascorbic and organic acids) and potential inhibitory factors (polyphenols and fibers). This duality is critical for interpreting the discrepancy between in vitro and human findings. The ascorbic acid (present at 163.8–288.9 mg/100 g dw) [45,47] reduces ferric iron (Fe^3+^) to ferrous iron (Fe^2+^), enhancing solubility at intestinal pH. Citric and malic acids chelate iron, maintaining solubility and preventing precipitation [66,67]. These effects are well documented for isolated organic acids and have been demonstrated in BFP-fortified food matrices in vitro [64,65,67]. BFP also contains polyphenols (proanthocyanidins, flavonol glycosides, hydroxycinnamic acid derivatives) that may inhibit non-heme iron absorption through chelation and precipitation [16,17,52]. In the Caco-2 cell model, Khoja et al. [66] observed that while BFP demonstrated the highest iron bioavailability among tested ingredients, the presence of polyphenols may have attenuated the full enhancing potential of ascorbic acid. Additionally, the fiber content of BFP (45.8–56.62 g/100 g dw) may bind iron and reduce its bioaccessibility, though the net effect of fiber on iron absorption is complex and depends on fiber type and food matrix [40,65]. The net effect of BFP on iron bioavailability depends on the relative concentrations of enhancers and inhibitors, which vary with provenance and processing. In food-to-food fortification studies, the enhancing effects of ascorbic and organic acids appear to dominate, resulting in increased iron bioaccessibility [40,65]. However, in the human intervention trial by Evang et al. [79], the inhibitory effects of dietary phytates and polyphenols from the subjects’ habitual diet may have outweighed BFP’s enhancing effects, contributing to the lack of significant improvement in iron status.

**Figure 2:**
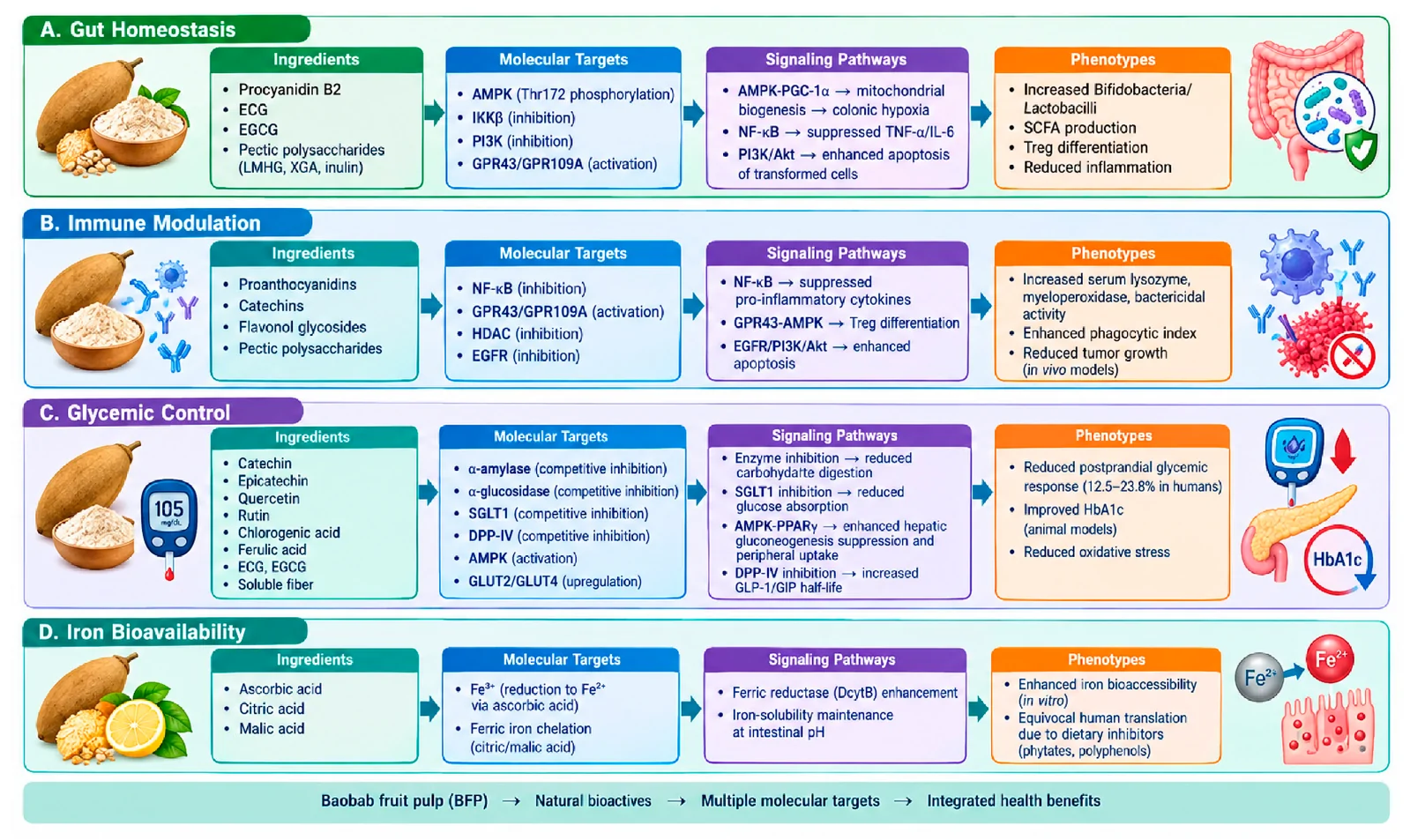
Integrated mechanism pathway diagram depicting “Ingredients → Molecular Targets → Signaling Pathways → Phenotypes” for BFP-mediated health benefits across four therapeutic domains. Solid arrows denote mechanisms supported by direct BFP-specific evidence (e.g., α-amylase and α-glucosidase inhibition, iron bioaccessibility enhancement, fiber fermentation in in vitro models). Dashed arrows denote mechanisms inferred from structurally similar compounds in other sources (e.g., AMPK-PGC-1α activation, NF-κB inhibition, PI3K/Akt modulation, SGLT1 inhibition, GLUT2/GLUT4 modulation, DPP-IV inhibition, GPR43/GPR109A activation). None of the pathways shown has been confirmed in human intervention studies. The graphical layout of this figure was prepared with assistance from ChatGPT (version 5.6), based on the authors’ original concept and idea.

The health-promoting effects of BFP are mediated by a diverse range of bioactive compounds that interact with multiple molecular targets and signaling pathways. Figure 2 summarizes the proposed mechanisms linking BFP constituents to physiological outcomes across four major therapeutic domains: gut homeostasis, immune modulation, glycemic control, and iron bioavailability. This integrated pathway highlights how the combined actions of polyphenols, pectic polysaccharides, organic acids, and other bioactive components may contribute to the multifaceted health benefits of BFP.

**Figure 3:**
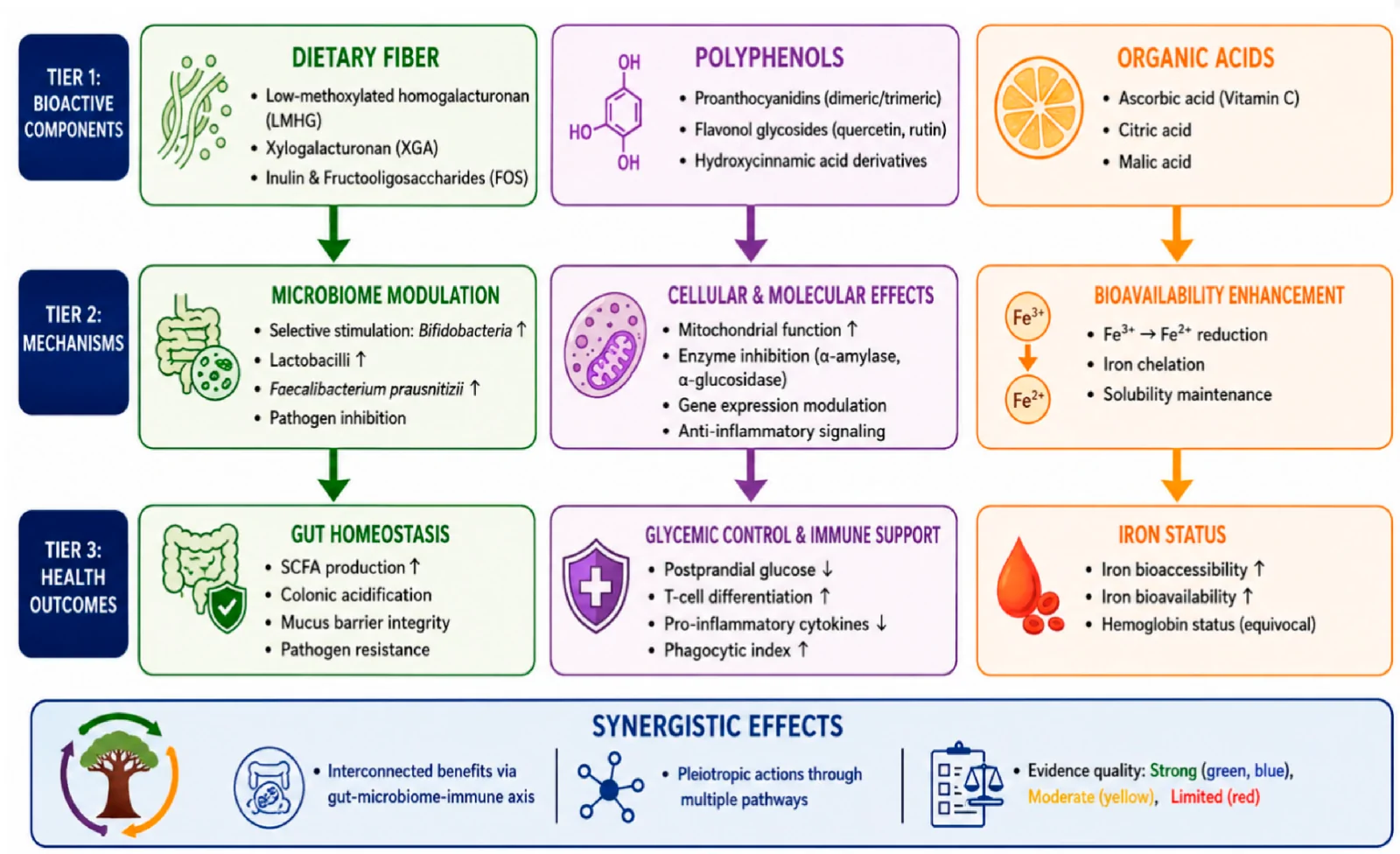
Mechanisms of action for baobab fruit pulp (BFP)-mediated health benefits. Pathways supported by direct BFP-specific evidence are distinguished from those inferred from other polyphenol or fiber sources. Direct evidence exists for α-amylase and α-glucosidase inhibition, iron bioaccessibility enhancement in food matrices, and fiber fermentation in in vitro gut models. All other mechanisms shown, including AMPK-PGC-1α-mediated mitochondrial effects, NF-κB and PI3K/Akt modulation, SGLT1 inhibition, GLUT2/GLUT4 modulation, DPP-IV inhibition, and GPR43/GPR109A-mediated Treg differentiation, are inferred from studies on structurally similar compounds and have not been demonstrated with BFP-specific preparations. No mechanism has been confirmed in human intervention studies. The graphical layout of this figure was prepared with assistance from ChatGPT (version 5.6), based on the authors’ original concept and data.

Van der Merwe [67] found that the inclusion of BFP powder in pearl millet porridge, particularly at a 1:10 (*w*/*w*) baobab:pearl millet ratio, improved iron bioaccessibility. Gabaza et al. [65] found that food-to-food fortification of maize, red sorghum, and white finger millet increased iron bioaccessibility. Similarly, the food-to-food fortification of maize-based whole-grain porridge with BFP enhanced the in vitro bioaccessibility of iron [64]. They determined that the effects of BFP were enhanced in the presence of higher quantities of available iron. The observed trend was also attributed to the high content of organic acids in baobab, particularly ascorbic, citric, and malic acids, which enhance the iron bioaccessibility [65,67]. This is consistent with the findings of Kim et al. [114], who observed that ascorbic acid can offset the inhibitory effects of dietary polyphenols on iron absorption. To a large extent, this explains why BFP significantly increased iron bioavailability compared with fenugreek and moringa [66]. In that study, the human Caco-2 cell line was used to evaluate iron bioavailability from fenugreek sprouts, seeds, baobab, and moringa in an in vitro simulated peptic–pancreatic digestion experiment. Iron in the ferric state is insoluble at a pH above 3, and as such, its reduction by ascorbic acid from the ferric (Fe^3+^) to ferrous (Fe^2+^) state increases its bioavailability at the physiological pH of the intestines [65]. Nevertheless, there is a need to support this argument using in vivo findings on the effects of BFP on iron bioaccessibility. Figure 3 provides an integrated overview of the proposed mechanisms by which BFP components exert their pleiotropic health benefits, linking specific bioactive compounds to physiological outcomes via defined molecular and cellular pathways.

Evang et al. [79] conducted a single-blind randomized controlled trial involving healthy schoolchildren aged 6–12 years (*N* = 58; intervention *n* = 29, control *n* = 29) with hemoglobin < 12.2 g/dL. The intervention group received a school meal with a BFP-supplemented drink, while the control group received the same meal with an isoenergetic drink without BFP for 12 weeks. Iron absorption was not directly measured in this trial. Iron status was assessed using hemoglobin, serum ferritin, and soluble transferrin receptor (sTfR) levels. Supplementation with BFP increased hemoglobin levels in the intervention group but did not produce significant improvements in serum ferritin or sTfR levels [79]. Several factors may explain this discrepancy between in vitro findings (bioaccessibility, Caco-2 uptake) and human iron status outcomes: (i) the inhibitory effects of concomitant dietary factors (phytates, other polyphenols) may outweigh BFP’s enhancing effects in real-world dietary contexts; (ii) the intervention duration (12 weeks) may be insufficient for measurable changes in iron stores, particularly in populations with adequate baseline iron status; (iii) the baseline iron status of the study population (mild anemia, hemoglobin < 12.2 g/dL) may require more intensive or prolonged intervention; (iv) differential bioavailability between BFP samples of different provenance (variation in ascorbic acid content ranges from 163.8 to 288.9 mg/100 g) may affect outcomes [47]; and (v) the intervention’s failure to align with a meal known to strongly inhibit iron absorption (e.g., high-phytate meals) may have limited the observable enhancing effect of ascorbic acid from BFP [99]. As noted above, a further consideration is that the hemoglobin response may reflect the combined effect of several nutrients in BFP and the accompanying meal rather than enhanced iron absorption specifically, and the absence of inflammation-adjusted ferritin data limits interpretation of the null ferritin finding. These human findings highlight a critical discrepancy: robust in vitro evidence for increased bioaccessibility and Caco-2 cell uptake does not necessarily translate to improved iron status in humans. This discrepancy may reflect the complex interplay of dietary inhibitors, individual variability in iron absorption capacity, and the limitations of short-term intervention duration for detecting changes in iron stores. Further research is warranted to address these challenges and to better understand the factors that moderate BFP’s iron-enhancing effects in real-world dietary contexts.

### 4.5. Caveats and Limitations in Mechanistic Interpretation

Several important caveats must be considered when interpreting the mechanistic evidence presented in this review:

#### 4.5.1. Distinction Between BFP-Specific and Extrapolated Evidence

Throughout this review, we have distinguished between: (a) mechanisms that have been directly demonstrated using BFP extracts, BFP-isolated compounds, or BFP-treated experimental models (BFP-specific evidence); and (b) mechanisms that are proposed based on the presence of known bioactive compounds in BFP and their established activities in other contexts (extrapolated mechanisms). Table 10 summarizes this distinction for each health theme.

#### 4.5.2. The Challenge of Complex Mixtures

BFP is a complex mixture of fibers, polyphenols, organic acids, and other phytochemicals [16,17,18,39,40]. When whole BFP extracts are used in experimental models, it is often impossible to attribute observed effects to specific compounds or compound classes. Foltz et al. [42] demonstrated prebiotic effects of whole BFP, but did not isolate whether these effects were primarily driven by pectic polysaccharides, polyphenols, or synergistic interactions between components. Similarly, the glycemic effects observed by Coe et al. [77] and Rita et al. [78] were attributed to both polyphenols and soluble fiber, but the relative contribution of each component remains unclear. Disentangling the contributions of individual BFP components to observed health effects is a critical research priority.

#### 4.5.3. Translation from In Vitro to In Vivo

Many of the mechanistic studies cited in this review were conducted in vitro using isolated enzymes, cell lines, or fermentation models. While these models provide valuable mechanistic insights, they do not account for the complexities of the in vivo environment, including: (i) bioavailability and metabolism of BFP compounds in the gastrointestinal tract; (ii) interactions with other dietary components; (iii) inter-individual variability in gut microbiota composition and metabolic capacity; and (iv) the influence of host physiology and genetics.

### 4.6. Limitations on Methodological and Evidence-Based

This review has several methodological limitations that should be acknowledged. First, while we conducted a systematic search following PRISMA guidelines, the review was not registered in a prospective registry (e.g., PROSPERO), and a formal risk-of-bias assessment for individual studies was not performed. Second, the heterogeneity in experimental designs, outcome measures, and BFP preparations across studies limited the feasibility of quantitative synthesis (meta-analysis). Third, the search was limited to peer-reviewed literature, and grey literature was not included, which may have introduced publication bias. Fourth, the evidence base is geographically concentrated in East and West Africa, with limited representation from Central and Southern African regions. Beyond methodological constraints, the evidence base for BFP health effects has inherent limitations that should be considered when interpreting the findings of this review. First, there is a predominant reliance on in vitro and animal model data, with limited human intervention studies available for comprehensive analysis.

A fundamental limitation of this review is the absence of a formal risk-of-bias assessment for included studies. Methodological quality domains, including randomization procedures, allocation concealment, blinding, handling of incomplete outcome data, and selective reporting, were not systematically evaluated using validated tools such as Cochrane RoB 2 for randomized trials or SYRCLE’s risk-of-bias tool for animal studies [121,122]. Consequently, the evidence tiers and overall evidence status assigned in this review reflect only the study design and should not be interpreted as judgments of methodological quality. The conclusions regarding the strength of evidence for BFP health effects are, therefore, tentative and require confirmation through future systematic reviews with formal risk-of-bias assessment. The translation of findings from cell cultures, fermentation models, and rodent studies to human populations remains uncertain. Second, there is substantial heterogeneity in the BFP preparations used across studies, including whole pulp, powder, aqueous extracts, and organic solvent extracts, which may differ substantially in composition and bioactivity. Third, most animal studies have used single-dose or short-term intervention protocols that do not reflect chronic consumption patterns in humans. Fourth, human studies are limited to healthy, normoglycemic adults (glycemic studies) and schoolchildren with mild anemia (iron studies), with no data available for diabetic, prediabetic, or other target populations. Fifth, the absence of systematic reviews and meta-analyses at the highest level of evidence represents a significant gap.

**Figure 4:**
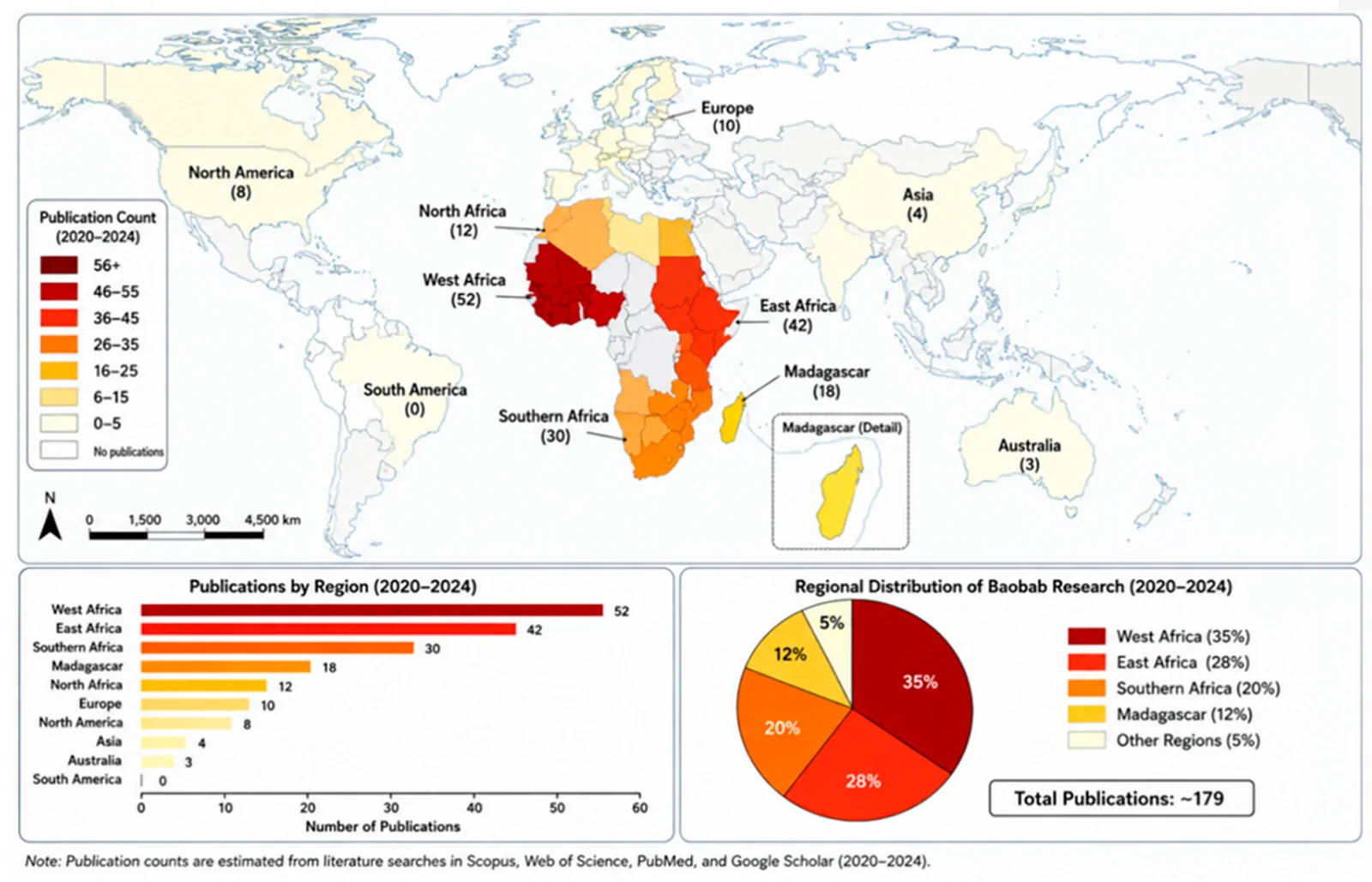
Geographic distribution of Baobab research publications by region.

Based on the identified evidence gaps, several priorities for future primary research emerge. Geographic expansion of baobab research to include under-studied African regions (Central and Southern Africa) is needed to understand provenance-specific variation in composition and bioactivity. Standardization of outcome measures across studies, including consistent definitions of iron bioaccessibility, bioavailability, and absorption, is essential to enable meaningful comparisons and future meta-analyses. Longer-term human intervention studies with clinically meaningful endpoints (HbA1c, ferritin, infection incidence) are needed to assess the translational relevance of promising in vitro and animal findings. Figure 4 illustrates the geographic distribution of baobab research publications, highlighting the concentration of evidence in West and East Africa while revealing significant research gaps in Central Africa and other regions. This geographic disparity underscores the need for expanded research efforts in understudied areas to ensure a comprehensive understanding of baobab’s nutritional and therapeutic potential across its full distribution range.

## 5. Challenges, Sustainability, and Future Research Priorities

Beyond biological efficacy and consumer acceptance, the realization of BFP’s nutritional therapeutic potential depends on sustainable production systems, ethical sourcing, and product integrity. This section addresses the sustainability, commercial, and quality challenges that affect the availability, consistency, and safety of BFP for both research and consumer applications.

### 5.1. Research Priorities

Based on the evidence gaps identified in the previous Section 4, several research priorities emerge for advancing the evidence base on BFP health benefits:

Clinical translation: Well-designed human intervention trials with clinically meaningful endpoints (e.g., HbA1c, ferritin, infection incidence, gut microbiome composition) are needed across diverse populations, including diabetic and pre-diabetic individuals, children with anemia, and elderly populations with gut dysbiosis. A randomized placebo-controlled trial investigating the effects of baobab fruit powder on gut and cardiometabolic health in obesity is currently underway and will provide valuable clinical evidence [123]. Briefly, wild harvesting of BFP supports rural livelihoods but requires sustainable management to ensure long-term availability of raw material for research and commercial applications [124]. Climate vulnerability and the 25-year juvenile period before fruiting [31] pose risks to supply continuity, potentially affecting the availability of BFP for clinical research. Benefit-sharing arrangements (e.g., Fair Trade certification) and protection of traditional knowledge under the Nagoya Protocol [25] are relevant to the ethical sourcing of BFP for clinical trials, though detailed discussion of these topics is beyond the scope of this review.

Mechanistic depth: Studies should isolate specific BFP compounds (procyanidins, catechins, pectic polysaccharides) and evaluate their relative contributions to observed health effects using pathway-specific assays (e.g., phosphorylation-specific Western blots, pathway inhibitors, reporter gene assays). The relative contribution of fiber vs. polyphenols vs. organic acids to gut homeostasis and glycemic regulation requires systematic investigation.

Bioavailability optimization: Research should examine how processing methods, food matrix interactions, and dietary context (e.g., phytate content, meal composition) affect the bioaccessibility and bioavailability of BFP bioactive compounds. Pharmacokinetic studies of BFP polyphenols following oral consumption are currently absent and urgently needed.

Consumer acceptance studies: Multi-country consumer surveys, sensory evaluation studies, and willingness-to-pay analyses are needed to inform product development strategies for different market segments (e.g., African vs. European consumers, first-time vs. experienced consumers).

### 5.2. Transitional Barriers

Aside from the biological efficacy, the translation of BFP into evidence-based nutritional applications depends on several practical factors that are briefly summarized here. BFP is commercially available as powder, beverages, energy bars, fermented dairy products, and bakery ingredients, and has been approved as a novel food ingredient in the European Union and the United States [20,24]. Consumer acceptance studies indicate that sensory attributes, particularly the balance between tartness and sweetness, are the strongest predictors of overall liking, and that prior familiarity with baobab increases acceptance [125]. Consumer segmentation analyses have identified distinct low-liking and high-liking groups with differing willingness to pay [125]. In African markets, awareness, availability, affordability, and product quality influence utilization intensity [126]. Product integrity remains a concern, with adulteration rates of 10–30% reported in commercial BFP powder samples, primarily with cereal flours [127]. Sustainable sourcing must also address pressure from wild harvesting, the long juvenile period before fruiting, and climate vulnerability [124,128]. These factors do not alter the mechanistic evidence assessed in this review but are relevant to whether promising preclinical findings can be translated into real-world nutritional applications. A detailed treatment of market dynamics, certification schemes, and benefit-sharing arrangements is beyond the scope of this review and is available elsewhere [22,25,26].

Based on the evidence gaps identified in this review, Table 11 provides a consolidated overview of the strategic research priorities outlined above, including the methodological approaches and expected outcomes for each priority area.

### 5.3. Sustainability Consideration

BFP is currently harvested predominantly from wild baobab populations, and sustainable management is needed to ensure long-term availability of raw material for research and commercial applications [121]. The 25-year juvenile period before fruiting [28] limits the scalability of cultivation-based production, and climate change poses additional risks to natural populations [123]. Detailed discussion of certification schemes, access and benefit-sharing arrangements, and commercial scaling is beyond the scope of this review and is available elsewhere [21,119,122].

## 6. Conclusions

This structured narrative review evaluates the evidence for proposed health benefits of baobab (*Adansonia digitata* L.) fruit pulp (BFP) across four domains: gut homeostasis, immune modulation, glycemic control, and iron bioavailability. BFP contains fermentable fibers (low-methoxylated homogalacturonan, xylogalacturonan, and possibly inulin and fructooligosaccharides), polyphenols (proanthocyanidins, flavonol glycosides, hydroxycinnamic acid derivatives), and organic acids (ascorbic, citric, and malic), which underpin the mechanisms examined in this review. The evidence base is predominantly preclinical. Tier 3 (in vitro) and Tier 2 (animal) studies report α-amylase and α-glucosidase inhibition, iron bioaccessibility enhancement, and selective stimulation of gut microbiota in fermentation models; the methodological quality of these studies was not assessed. However, only six human intervention studies were identified, all limited to glycemic response in healthy, normoglycemic adults, with one iron status trial in schoolchildren. No human studies have examined gut microbiota modulation, immune outcomes, or effects in diabetic, pre-diabetic, or other target populations. Clinical efficacy has therefore not been established, and BFP cannot currently be described as having confirmed prebiotic, immunomodulatory, or therapeutic properties. Four major limitations define the current evidence base: (i) the absence of adequately powered human intervention trials with clinically meaningful endpoints; (ii) the lack of pharmacokinetic data on BFP polyphenols in humans; (iii) the unresolved contribution of individual BFP components (fibers, polyphenols, organic acids) to observed effects; and (iv) insufficient standardization of BFP preparations and quality control, which limits comparability across studies. A further limitation is the absence of a formal risk-of-bias assessment, meaning the evidence tiers presented reflect study design only and cannot distinguish between high-quality and low-quality studies of the same design type. BFP nonetheless shows potential as a sustainable, nutrient-dense food ingredient, but this potential should be interpreted cautiously. Practical barriers to translation, including raw-material variability, adulteration, sustainable sourcing, and sensory acceptance, are relevant but were not systematically assessed in this review. Priority should now be given to randomized, well-controlled human trials with clinically meaningful endpoints, standardized BFP preparations, and systematic investigation of real-world dietary contexts, before evidence-based dietary recommendations or health claims can be made.

## Figures and Tables

**Table 3:** Summary of experimental evidence for the effects of baobab fruit pulp (BFP) on gut homeostasis.

Type	Source	Experimental Model	Key Findings	Evidence Level	Reference
In vitro	Döhler GmbH (Darmstadt, Germany)	Human fecal microbiota batch incubation with BFP (0–48 h); qPCR quantification of bacterial taxa; SCFA and lactate measurement	↑ colonic acidification; donor-dependent ↑ in *Bifidobacteria*, *Lactobacilli*, and *Firmicutes*	IV	[42]
In vitro	Wukari, Nigeria	*Lactobacillus rhamnosus* GG (LGG) cultivation in BFP-supplemented medium; LGG viability in white pan bread and simulated GI passage.	↑ LGG viability during cultivation (3.05%) and in bread (2.96%); ↑ viability during intestinal passage (3.05–11.48%).	IV	[69]
In vitro	Nexira (Rouen, France)	SHIME^®^ gut model simulator; 3-week BFP and Arabic gum supplementation; 16S rRNA sequencing	↑ *Bifidobacteriaceae*, *Barnesiellaceae*, *Akkermansiaceae*, *Christensenellaceae* (distal colon); ↑ SCFA, lactate, spermidine	IV	[43]
In vitro	South Africa [University of Johannesburg]	Synbiotic beverage development with BFP and probiotic lactic acid bacteria	BFP exhibited prebiotic and antimicrobial effects; supported LAB growth	IV	[41]
In vivo	Zimbabwe [University of Zimbabwe]	Mutandabota production (milk:BFP:sugar, 15:2.5:1); *L. rhamnosus* yoba fermentation	BFP promoted probiotic growth; inactivated *L. monocytogenes*, *E. coli*, *Salmonella* spp.; pH 3.5	III	[70,71]

↑ indicates an increase in the corresponding parameter.

**Table 4:** Biological effects of baobab (*Adansonia digitata* L.) fruit pulp.

Type	Source	Experimental Model	Key Findings	Evidence Level	Reference
In vivo	Free Trade Egypt Co. (Alexandria-Desert Road, Alexandria, Egypt)	Nile tilapia fed BFP-supplemented diet (0–4 g/kg) for 2 months	↑ serum lysozyme, myeloperoxidase, bactericidal activities; ↑ hepatic CAT, SOD, T-AOC; ↓ MDA; ↑ *inf-γ1, lyz, il-1β, il-10*, hepcidin	III	[72,73]
In vivo	Makkah, Saudi Arabia	Wistar albino rats (125–150 g); CCl_4_-induced hepatotoxicity; BFP extract (1 mg/kg oral, 15 days)	Normalized serum ALT, AST, ALP; hepatoprotective effects attributed to triterpenoids, β-sitosterol, β-amyrin palmitate, ursolic acid	III	[74]
In vivo	Mandu, Madhya Pradesh, India	Wistar rats; SRBC-induced DTH response; methanolic BFP extract (250 or 500 mg/kg)	↑ T-cell stimulation; ↑ SRBC-induced antibody titer; ↑ phagocytic index	III	[68]
In vitro	Sudan	Human cancer cell lines: HCT116 (colon), MCF-7 (breast), OVCAR-3/4 (ovarian); polar and non-polar BFP extracts	Polar fiber extracts inhibited HCT116 and MCF-7 cells; modulated cell cycle, apoptosis, and proliferation pathway genes	IV	[75]
In vivo	Sudan	BALB/C mice; Ehrlich Ascites Carcinoma (EAC) model; BFP seed/fruit extract (300 mg/kg, 14 days)	Modulated serum redox state; restored α-l-fucosidase and arginase; attenuated *p53* and *Bcl-2* expression; ↓ tumor growth	III	[76]
In vivo	Dodoma, Tanzania	BALB/c mice (34.7 ± 0.5 g); BFP pectin extract (8.7 mg/mL); forced swim test (FST)	Significant antidepressant-like effect in FST; no effect in OFT or LBT	III	[44]

↑ indicates an increase, while ↓ indicates a decrease in the corresponding parameter.

**Table 5:** Summary of experimental evidence of the glycemic-regulating effects of baobab fruit pulp (BFP).

Type	Source	Experimental Model	Key Findings	Evidence Level	Reference
In vivo	Khartoum, Sudan	Albino rats (250–300 g, *N* = 70, *n* = 10); alloxan-induced diabetes; BFP (500 mg/kg oral); metformin control	↓ blood sugar levels; ↓ TNF-α, IL-6, and IL-10 levels	IV	[49]
In vivo	Khartoum state, Sudan	Sprague Dawley rats (150–200 g, *N* = 36, *n* = 6); alloxan-induced diabetes; BFP (150 or 300 mg/kg)	Hypoglycemic effect; restored kidney histology; ↓ ALP, ALT, AST	III	[50]
In vivo	Min Vita, London, UK	Healthy female subjects (*n* = 9); single-blind, repeated-measures; white bread + BFP (18.5 or 37 g)	Dose-dependent ↓ glycemic response: low dose 12.5%, high dose 23.8%	II	[77]
In vitro	Ngaoundéré, Cameroon	Enzyme inhibition assays: α-amylase, α-glucosidase, ACE, HMG-CoA reductase, pancreatic lipase; SW-872 liposarcoma cells	Concentration dependent inhibition of α-amylase (IC_50_—9.97 mg/mL) and α-glucosidase (IC_50_—0.64 mg/mL)	III	[57]
In vivo	Luanda, Angola	Healthy adults (*N* = 31, intervention *n* = 15); glucose + BFP vs. glucose control	↓ Glycemic iAUC; ↓ glucose maximum concentration; 18.7% reduction (*p* < 0.05)	II	[78]
In vivo	Kordofan State, West Sudan	Wistar rats (140–200 g, *N* = 42, *n* = 6); HS/HFD-induced metabolic syndrome; BFP (200, 400, 800 mg/kg)	↓ AIP; ↓ creatinine/urea; anti-inflammatory, antilipidemic, hypoglycemic activities; restored organ histology	III	[8]

↓ indicates a decrease in the corresponding parameter.

**Table 6:** Summary of experimental evidence for the iron bioavailability-enhancing effects of baobab fruit pulp (BFP).

Type	Source	Experimental Model	Key Findings	Evidence Level	Reference
In vitro	Dakar, Senegal	Pearl millet porridge formulations with BFP, moringa, roselle; iron bioaccessibility assay.	↑ iron bioaccessibility at 1:10 (BFP: pearl millet) ratio	IV	[67]
In vitro	Victoria Falls, Zimbabwe	Fermented cereals: maize, red sorghum, white finger millet; BFP-enriched vs. non-enriched	↑ iron bioaccessibility in BFP-enriched cereals	IV	[65]
In vitro	Durban, South Africa	Wholegrain maize-based porridge; BFP and/or moringa fortification	↑ percentage and total bioaccessible iron and zinc	IV	[64]
In vitro	Aduna Ltd., London, UK	Caco-2 cell model; simulated peptic–pancreatic digestion; BFP vs. fenugreek vs. moringa	BFP showed highest percentage iron bioavailability; attributed to ascorbic, citric, malic acids	IV	[66]
In vivo	Kenya	Schoolchildren (6–12 years, *N* = 58; intervention *n* = 29, control *n* = 29); BFP-supplemented drink; 12 weeks	↑ hemoglobin levels; no significant change in ferritin or sTfR; effect attenuated by dietary phytates/polyphenols	II	[79]

↑ indicates an increase.

**Table 7:** Concentrations of key polyphenols in BFP and composition with reported effective concentrations.

Polyphenol	Concentration in BFP (mg/g dw)	Estimated Intake per Serving (10–20 g BFP)	Reported Effective Concentration (IC_50_/EC_50_)	Reference for Bioactivity	Status (Human Exposure Not Established)
Procyanidin B2	0.5–2.0 mg/g [17,18,50]	5–40 mg	10–50 µM (AMPK activation)	[97]	Not established; requires PK data
Catechin	1–5 mg/g (estimated) [18,50]	10–100 mg	20–100 µM (α-amylase inhibition)	[17,57]	Not established; requires PK data
Epicatechin	1–5 mg/g (estimated) [18,50]	10–100 mg	20–100 µM (α-amylase inhibition)	[17,57]	Not established; requires PK data
Chlorogenic acid	0.5–1.5 mg/g [17]	5–30 mg	50–200 µM (α-glucosidase inhibition) [17]	[17]	Not established; requires PK data
Ferulic acid	0.2–0.8 mg/g [16]	2–16 mg	50–200 µM (α-glucosidase inhibition) [17]	[17]	Not established; requires PK data
Quercetin/Rutin	Not quantified (major by HPLC) [16,51]	Unknown	10–100 µM (enzyme inhibition) [57]	[57]	Not established; requires quantitative and PK data

Abbreviations: dw: dry weight; IC_50_: half-maximal inhibitory concentration; EC_50_: half-maximal effective concentration. PK: pharmacokinetic. Note: In vitro effective concentrations are not directly comparable to human target tissue exposure. Bridging this gap requires pharmacokinetic data on absorption, first-pass metabolism, colonic microbial degradation, plasma protein binding, and tissue distribution, none of which are currently available for BFP polyphenols.

**Table 8:** Evidence status for proposed glycemic mechanisms of BFP.

Proposed Mechanism	BFP-Specific Evidence	Evidence Source	Descriptive Status (Quality Not Assessed)
α-amylase inhibition	Direct	BFP extracts inhibit enzyme (IC_50_ 9.97 mg/mL) [57]; docking studies with BFP compounds [17]	Tier 3 evidence available (*n* = 2); quality not assessed
α-glucosidase inhibition	Direct	BFP extracts inhibit enzyme (IC_50_ 0.64 mg/mL) [57]; docking studies with BFP compounds [17]	Tier 3 evidence available (*n* = 2); quality not assessed
SGLT1 competitive inhibition	None	No BFP-specific studies; inferred from green tea polyphenol studies [59]	No BFP-specific evidence; inferred only
GLUT2/GLUT4 modulation	None	No BFP-specific studies; inferred from general polyphenol literature [58]	No BFP-specific evidence; inferred only
DPP-IV inhibition	Preliminary	One in vitro study with BFP extracts [57]; requires replication	Tier 3 evidence available (*n* = 1); quality not assessed

**Table 9:** Distinction of iron-related findings by experimental model and parameter measured.

Model Type	Parameter Measured	Key Findings	Translational Relevance
Simulated digestion (in vitro)	Iron bioaccessibility	BFP increases iron release from food matrix [64,65,67]	Indicates potential for enhanced absorption; does not account for intestinal uptake, transport, or systemic regulation
Caco-2 cell model	Iron uptake and bioavailability	BFP shows highest iron bioavailability among tested ingredients [66]	Approximates intestinal uptake; does not account for systemic factors (hepcidin, iron stores, dietary interactions)
Human intervention	Iron absorption	Not directly measured	–
Human intervention	Iron status (hemoglobin, ferritin, sTfR)	Increased hemoglobin; no significant change in ferritin or sTfR [79]	Reflects long-term iron balance; influenced by multiple factors beyond single-meal absorption

**Table 10:** Evidence status for BFP mechanisms: Direct evidence vs. extrapolated mechanisms.

Health Theme	Proposed Mechanism	BFP-Specific Evidence Status	Extrapolated From	Descriptive Status (Quality Not Assessed)
Gut homeostasis	Polyphenol-mediated mitochondrial biogenesis (AMPK-PGC-1α)	Indirect—BFP polyphenols characterized; no direct in vivo tracking	General polyphenol literature [79,82]	No BFP-specific evidence; inferred only
Gut homeostasis	SCFA-mediated Treg differentiation (GPR43/109A)	Indirect—BFP increases SCFA production [40,58]; no direct Treg demonstration	General SCFA literature [89,90,91]	No BFP-specific evidence; inferred only
Glycemic control	α-amylase inhibition	Direct—BFP extracts inhibit enzyme (IC_50_ 9.97 mg/mL) [57]; docking studies with BFP compounds [17]	BFP-specific	Tier 3 evidence available (*n* = 2); quality not assessed
Glycemic control	α-glucosidase inhibition	Direct—BFP extracts inhibit enzyme (IC_50_ 0.64 mg/mL) [57]; docking studies with BFP compounds [17]	BFP-specific	Tier 3 evidence available (*n* = 2); quality not assessed
Glycemic control	SGLT1 competitive inhibition	None—no BFP-specific studies	Green tea polyphenol literature [59]	No BFP-specific evidence; inferred only
Glycemic control	GLUT2/GLUT4 modulation via AMPK-PPARγ	None—no BFP-specific studies	General polyphenol literature [58]	No BFP-specific evidence; inferred only
Glycemic control	DPP-IV inhibition	Preliminary—one in vitro study [57] requires replication	BFP-specific (preliminary)	Tier 3 evidence available (*n* = 1); quality not assessed
Immune modulation	NF-κB pathway inhibition	Indirect—BFP modulates immune genes [75]; no pathway-specific confirmation	General polyphenol literature [88,96]	No BFP-specific evidence; inferred only
Immune modulation	PI3K/Akt/EGFR pathway modulation	Indirect—BFP modulates apoptosis genes [75]; no pathway-specific confirmation	General polyphenol literature [91,92]	No BFP-specific evidence; inferred only
Iron bioavailability	Ascorbic acid-mediated Fe^3+^ → Fe^2+^ reduction	Direct—BFP organic acids enhance iron bioaccessibility in vitro [64,65,66,67]	BFP-specific	Tier 3 evidence available (*n* = 4); quality not assessed; human translation equivocal

**Table 11:** Strategic Research Priorities for BFP health benefits.

Priority Area	Research Questions	Methodological Approaches	Expected Outcomes
Clinical translation	Do BFP health benefits translate to clinically meaningful outcomes in target populations?	RCTs in diverse populations (diabetics, anemic children, elderly); ≥12-week interventions; clinically meaningful endpoints (HbA1c, ferritin, infection incidence)	Evidence-based dietary recommendations for specific population groups
Mechanistic depth	What is the relative contribution of fiber vs. polyphenols vs. organic acids?	Isolated compound studies; combination studies; pathway-specific assays (Western blots, inhibitors, reporter genes)	Identification of primary active compounds; standardized BFP preparations
Bioavailability optimization	How do processing and food matrix affect BFP bioactivity?	In vitro digestion models; in vivo pharmacokinetic studies; food matrix interaction studies	Optimized processing and formulation strategies
Consumer acceptance	What factors drive adoption of BFP products in different markets?	Multi-country consumer surveys; sensory evaluation; willingness-to-pay analyses	Market-informed product development strategies
Sustainable production	Can propagation and cultivation be optimized while preserving genetic diversity?	Germplasm characterization; breeding programs; agroforestry systems assessment; climate resilience studies	Scalable, sustainable supply chain models

## Data Availability

No new data were created or analyzed in this study. Data sharing is not applicable to this article.
